# Supplementary material for: Aperiodic approximants bridging quasicrystals and modulated structures
Source: Nat Commun. 2024 Jul 11;15:5742. doi: 10.1038/s41467-024-49843-4 (PMC11239952; doi:10.1038/s41467-024-49843-4)
Supplement: Supplementary file 1 — Supplementary Infomation [file 41467_2024_49843_MOESM1_ESM.pdf]

Supplementary Information

Aperiodic approximants bridging quasicrystals and modulated structures

Toranosuke Matsubara,[1] Akihisa Koga,[1] Atsushi Takano,[2]
Yushu Matsushita,[3] and Tomonari Dotera[4]

[1] *Department of Physics, Tokyo Institute of Technology, Meguro, Tokyo 152-8551, Japan*

[2] *Department of Molecular and Macromolecular Chemistry, Nagoya University, Nagoya, Aichi 464-8603, Japan*

[3] *Toyota Physical and Chemical Research Institute, Nagakute, Aichi 480-1192, Japan*

[4] *Department of Physics, Kindai University, Higashi-Osaka, Osaka 577-8502, Japan*

Contents

1. Substitution rules for the hexagonal metallic-mean tilings 3
2. Frequencies of the vertices 6
3. Honeycomb domain 8
4. Domain boundaries 11
5. Higher-dimensional representation 13
6. Phason flips 20
7. Lattice structure factors 23
8. Crystallographic description of the P31m particle system 26
9. Crystallographic description of the P31m polymer blend system 29
10. Pentagon tilings 31
11. Colloidal system 32
12. Atomic decorations 34

References 44

Supplementary Note 1 Substitution rules for the hexagonal metallic-mean tilings

The hexagonal metallic-mean tiling we propose in the main text is composed of large hexagons (L), parallelograms (P), and small hexagons (S). The length ratio is given by the metallic mean τ_k, with $\tau_k = (k + \sqrt{k^2+4})/2$. Extending the rule for the hexagonal golden-mean tiling [1], we propose the substitution rules for the metallic-mean tilings, which are explicitly shown in Suppl. Fig. 1. When the deflation rule is applied to an L tile, an

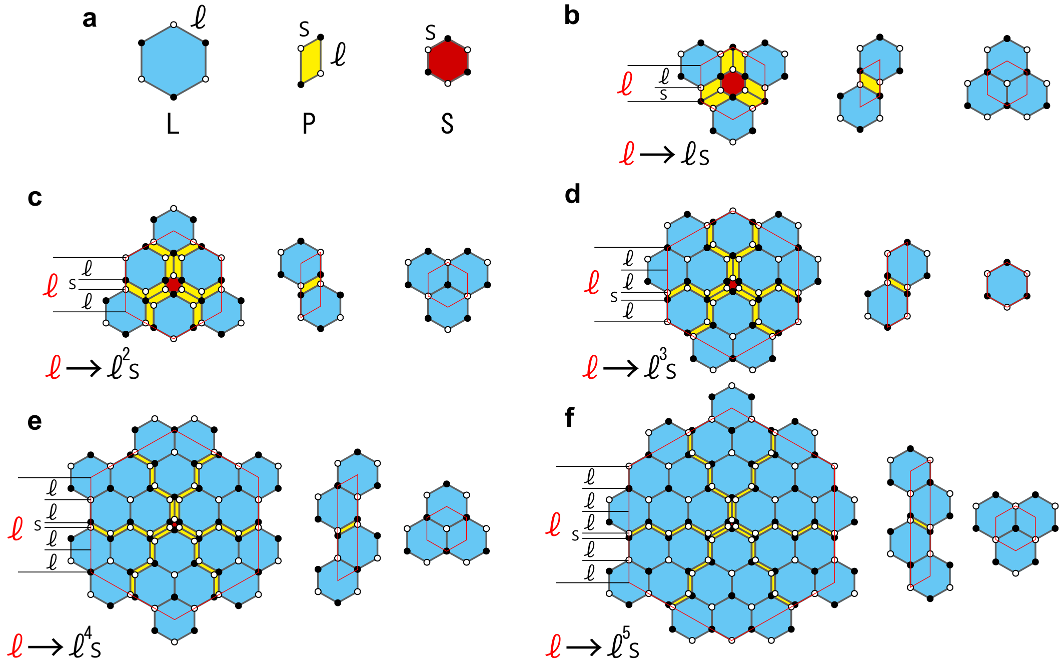

Supplementary Fig. 1. **Substitution rules for the hexagonal metallic-mean tilings. a** Large hexagon, parallelogram, and small hexagon. **b-f** Substitution rules for the golden-mean, silver-mean, bronze-mean, 4th metallic-mean, and 5th metallic-mean tilings.

S tile is generated at the center of the original L tile. Furthermore, six chains sharing a short edge of k P tiles are adjacent to the S tile and are located along six directions $(\cos(\pi i/3), \sin(\pi i/3))$ $(i = 0, 1, \cdots, 5)$. The rest region is filled by k^2 L tiles. As for a P tile, $k/3$ L tiles are generated along the longer edge of the original P tile, and one P tile is generated around the center. Note that there are two kinds of the P tiles: a P tile shown

in Suppl. Fig. 1a and its reflected tile ($\bar{\mathrm{P}}$ tile). We find that the P ($\bar{\mathrm{P}}$) tile appears for odd (even) k case when the substitution rule is applied to a P tile. An S tile is replaced to one L tile under one deflation operation. When the substitution rule is applied to an S tile, one L tile appears. More precisely, the substitution is classified by modulo 3 of k, as illustrated in Suppl. Fig. 1. Specifically, when $k \equiv 1$ and $k \equiv 2$ (mod 3), a site represented by an open or filled circle appears at the center, respectively, while no vertex is generated otherwise ($k \equiv 0$ mod 3). Since the arrangement of the resulting L tiles is uniquely determined, we are able to construct the substitution rule for the S tile for any given k. Moreover, we observe the same modulo 3 property for L and P tiles concerning corner sites. Thus, we can extend the substitution rule to encompass any metallic-mean tiling. These allow us to generalize the substitution rules for any metallic-mean tilings.

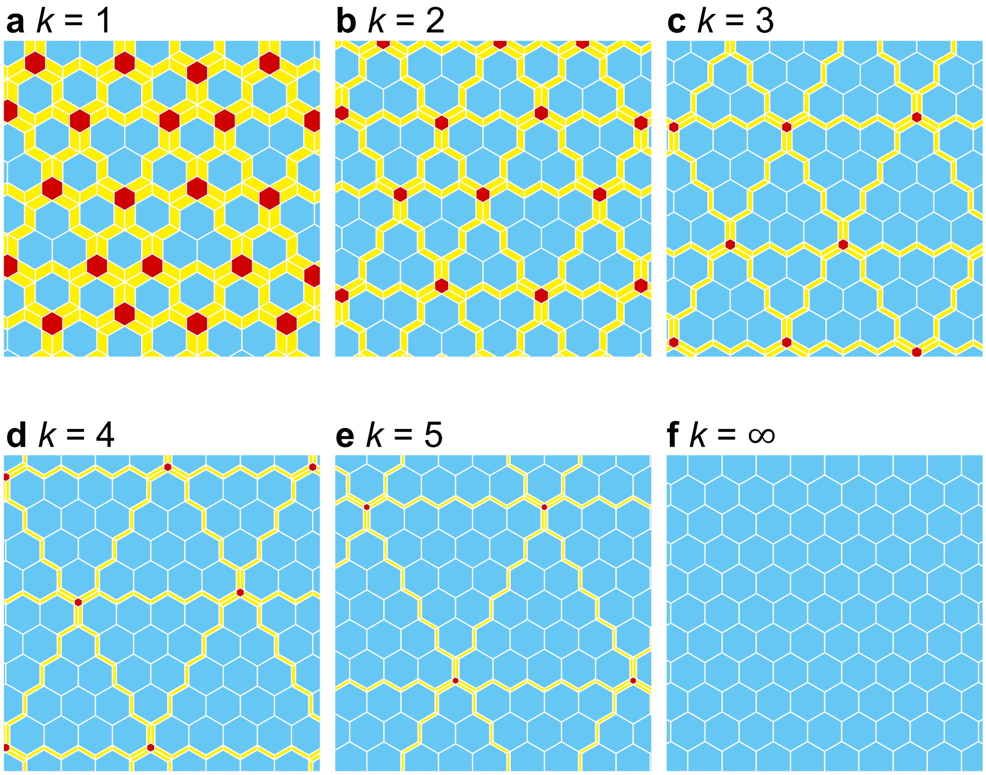

Supplementary Fig. 2. **Hexagonal metallic-mean tilings.** **a** Golden-mean tiling ($k = 1$) [1], **b** silver-mean tiling ($k = 2$) and **c** bronze-mean tiling ($k = 3$). **d** and **e** represent metallic-mean tilings with $k = 4$ and $k = 5$. **f** represents the honeycomb lattice with $k \to \infty$.

We obtain the hexagonal metallic-mean tilings, applying the substitution rule to a certain

tile iteratively. The metallic-mean tilings for $k = 1, 2, 3, 4, 5$, and the honeycomb lattice, which can be regarded as the tiling with $k \to \infty$, are shown in Suppl. Fig. 2. When one deflation operation is applied to the tilings, the number of tiles increases and the self-similar structure appears, which are shown in Suppl. Fig. 3a,b.

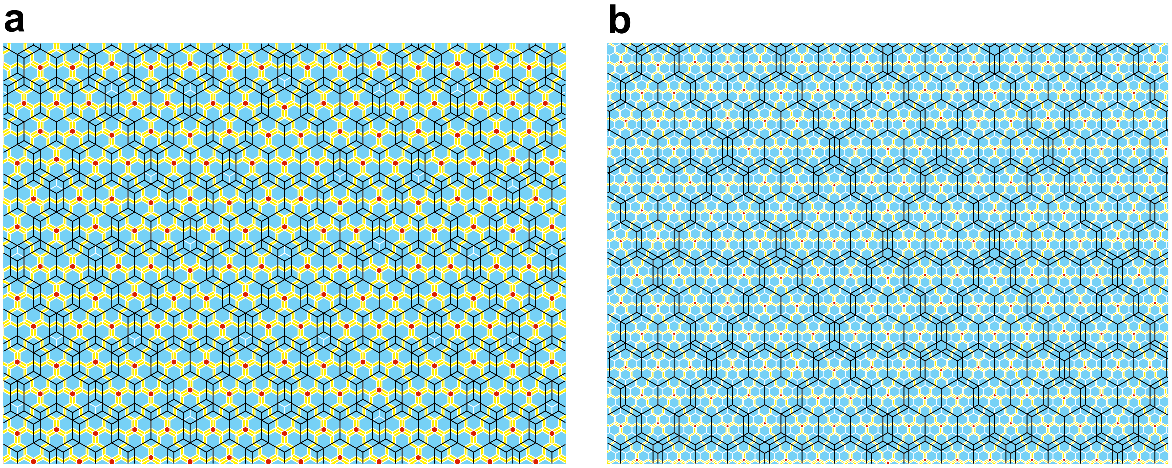

Supplementary Fig. 3. **Self-similarity of hexagonal metallic-mean tilings.** **a** Silver-mean tiling. **b** Bronze-mean tiling. The colored tilings are obtained by applying the deflation operation to the tilings shown as the black lines.

Supplementary Note 2 Frequencies of the vertices

We derive the frequencies of vertices for $k \neq 1$ (the frequencies for $k = 1$ have been given in Ref. [1]). In the case of $k \neq 1$, we could not find the F vertex shared by six P tiles ($f_{\mathrm{F}} = 0$). This is because vertices shared by two adjacent P tiles are always shared by the L or S tile according to the substitution rules for $k \neq 1$, as shown in Suppl. Fig. 1c-f. When one evaluates the frequencies for certain graphs such as vertices and domains, it is convenient to consider the ratio between numbers of tiles and vertices for the hexagonal metallic-mean tiling in the thermodynamic limit. Suppl. Fig. 1a clearly shows that the net numbers of sites in L, P, and S tiles are two, one, and two, respectively. Therefore, we obtain the ratio r_k as

$$r_k = 2f_{\mathrm{L}} + f_{\mathrm{P}} + 2f_{\mathrm{S}} = \frac{P_k}{\tau_k^2 + 6\tau_k + 1}, \tag{1}$$

where $P_k = 2\tau_k^2 + 6\tau_k + 2$. We first focus on the bipartite structure. The sublattice structures for the L, P, and S tiles are shown as the open and solid circles in Suppl. Fig. 1a, where these are referred to as A and B sublattices. By counting the net numbers of the site belonging to each sublattice in L, P, and S tiles, we obtain its frequencies as,

$$f_{\mathrm{A}} = \frac{1}{r_k}\left(f_{\mathrm{L}} + \frac{2}{3}f_{\mathrm{P}} + f_{\mathrm{S}}\right) = \frac{1}{2} + \frac{\tau_k}{P_k}, \tag{2}$$

$$f_{\mathrm{B}} = \frac{1}{r_k}\left(f_{\mathrm{L}} + \frac{1}{3}f_{\mathrm{P}} + f_{\mathrm{S}}\right) = \frac{1}{2} - \frac{\tau_k}{P_k}. \tag{3}$$

This naturally leads to the sublattice imbalance in the hexagonal metallic-mean tilings,

$$\Delta = f_{\mathrm{A}} - f_{\mathrm{B}} = \frac{1}{3 + \sqrt{k^2 + 4}}. \tag{4}$$

Since the sublattice A (B) is composed of C_1, C_2, and C_3 (C_0, D_0, D_1, and E) vertices, we obtain the following equations,

$$f_{\mathrm{A}} = f_{\mathrm{C}_1} + f_{\mathrm{C}_2} + f_{\mathrm{C}_3}, \tag{5}$$

$$f_{\mathrm{B}} = f_{\mathrm{C}_0} + f_{\mathrm{D}_0} + f_{\mathrm{D}_1} + f_{\mathrm{E}}. \tag{6}$$

In the tilings, two adjacent tiles share the edge, which is connected between the neighboring sites in A and B sublattices. Therefore, we obtain the equations for the total number of longer and shorter edges,

$$3f_{\mathrm{C}_1} + 2f_{\mathrm{C}_2} + f_{\mathrm{C}_3} = 3f_{\mathrm{C}_0} + 3f_{\mathrm{D}_0} + 2f_{\mathrm{D}_1} + 3f_{\mathrm{E}}, \tag{7}$$

$$f_{\mathrm{C}_2} + 2f_{\mathrm{C}_3} = f_{\mathrm{D}_0} + 2f_{\mathrm{D}_1} + 2f_{\mathrm{E}}, \tag{8}$$

where the left (right) hand side of the equations represents the total number of edges, which is expressed by the numbers of vertices belonging to the A (B) sublattice. According to the substitution rule, the C_3, D_1, and E vertices always appear around the S tile for $k \neq 1$. Therefore, these frequencies are then given as

$$f_{\mathrm{C}_3} = f_{\mathrm{D}_1} = f_{\mathrm{E}} = \frac{3f_{\mathrm{S}}}{r_k} = \frac{3}{P_k}. \tag{9}$$

From these equations, we obtain the exact frequencies of vertices in the hexagonal metallic-mean tilings as

$$f_{\mathrm{C}_0} = \begin{cases} 0 & (k=1) \\ \frac{1}{2} - \frac{7}{4\tau_k} + \frac{1}{2P_k}(27-7k) & (k \neq 1) \end{cases}, \tag{10}$$

$$f_{\mathrm{C}_1} = \frac{1}{2} - \frac{5}{4\tau_k} + \frac{5}{2P_k}(3-k), \tag{11}$$

$$f_{\mathrm{C}_2} = \frac{3}{2\tau_k} - \frac{3}{P_k}(4-k), \tag{12}$$

$$f_{\mathrm{C}_3} = \frac{3}{P_k}, \tag{13}$$

$$f_{\mathrm{D}_0} = \begin{cases} \frac{3}{4\tau_1^5} & (k=1) \\ \frac{3}{2\tau_k} - \frac{3}{P_k}(6-k) & (k \neq 1) \end{cases}, \tag{14}$$

$$f_{\mathrm{D}_1} = \frac{3}{P_k}, \tag{15}$$

$$f_{\mathrm{E}} = \begin{cases} \frac{3\sqrt{5}}{4\tau_1^5} & (k=1) \\ \frac{3}{P_k} & (k \neq 1) \end{cases}, \tag{16}$$

$$f_{\mathrm{F}} = \begin{cases} \frac{1}{4\tau_1^7} & (k=1) \\ 0 & (k \neq 1) \end{cases}. \tag{17}$$

The average of the coordination number is given by

$$\begin{aligned} z_k &= 3\sum_i f_{\mathrm{C}_i} + 4\sum_i f_{\mathrm{D}_i} + 5f_{\mathrm{E}} + 6f_{\mathrm{F}} \\ &= 3 + \frac{3}{2\tau_k} + \frac{3(k-3)}{P_k}. \end{aligned} \tag{18}$$

In the hexagonal metallic-mean tilings, the average of the coordination number depends on k. $z_k \to 3$ when the system approaches the honeycomb lattice $k \to \infty$.

Supplementary Note 3 Honeycomb domain

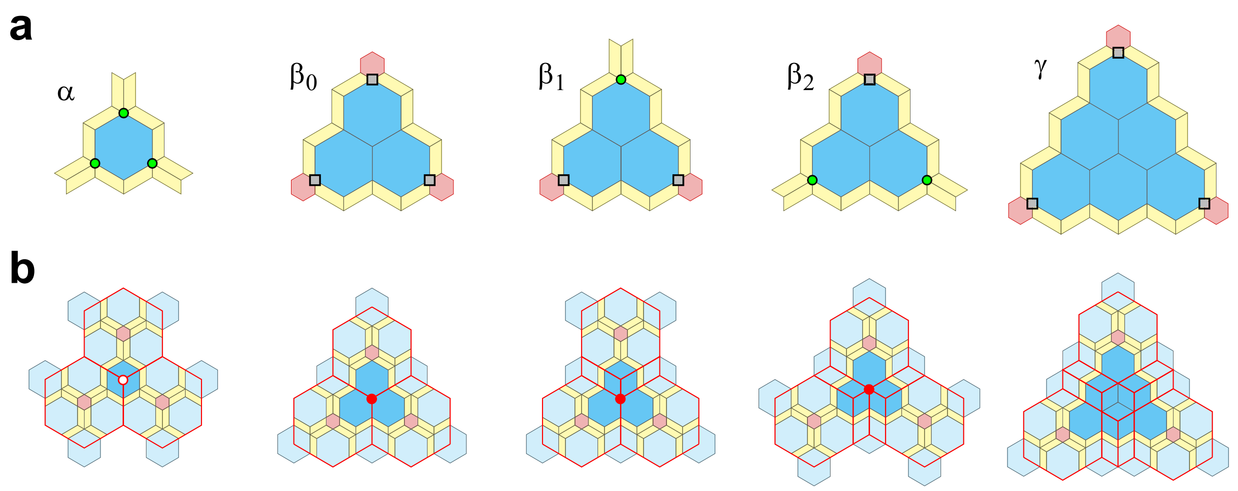

Supplementary Fig. 4. **Honeycomb domains.** **a** Dark blue hexagons represent $\alpha, \beta_0, \beta_1, \beta_2$, and γ domains in the hexagonal silver-mean tiling, which are bounded by some P and S tiles. Circles and squares indicate E and D_1 vertices at the corners of the honeycomb domains. **b** The red lines represent the results of the inflation operation applied to the tiles around the honeycomb domains shown in **a**. Red open (solid) circles at the vertices indicate the A (B) sublattice in the inflated tiling.

Here, we focus on the honeycomb domain in the hexagonal metallic-mean tilings with $k \neq 1$, which is composed of finite number of the L tiles and is bounded by the P and S tiles. As seen in Suppl. Fig. 2, in the hexagonal metallic-mean tiling with $k \neq 1$, there exist three kinds of domains composed of a_{k-1}, a_k or a_{k+1} L tiles, where $a_k = k(k+1)/2$. These are referred to as α, β and γ domains. Suppl. Fig. 4a shows α, β, and γ domains in the silver-mean tilings, as an example. We find that in the α domain, the E vertex shared by one L tile and four P tiles, which is shown as the circle, is located at each corner site. In the γ domain, the D_1 vertex shared by one L tile, two P tiles and one S tile, which is shown as the square, is located at each corner site. On the other hand, the β domains can be divided into the β_i ($i = 0, 1, 2$) domains, where i E vertices and $(3 - i)$ D_1 vertices are located at three corner sites, as shown in Suppl. Fig. 4a. The absence of the β_3 domains will be proved below.

To examine the frequency of each domain, we consider the substitution rule for the tiles. In Suppl. Fig. 4b, we show the tiling structure obtained by the inflation operation as the red lines. We find that the C_1, C_0, D_0, E vertices, and S tiles generated by an inflation operation are located at the center of the α, β_0, β_1, β_2, and γ domains. Therefore, we obtain the following equations as

$$f_\alpha = \frac{r_k f_{\mathrm{C}_1}}{\tau_k^2}, \tag{19}$$

$$f_{\beta_0} = \frac{r_k f_{\mathrm{C}_0}}{\tau_k^2}, \tag{20}$$

$$f_{\beta_1} = \frac{r_k f_{\mathrm{D}_0}}{\tau_k^2}, \tag{21}$$

$$f_{\beta_2} = \frac{r_k f_{\mathrm{E}}}{\tau_k^2}, \tag{22}$$

$$f_\gamma = \frac{f_{\mathrm{S}}}{\tau_k^2}, \tag{23}$$

where f_X is the ratio of the number of $X(=\alpha,\beta_i,\gamma)$ domains to the total number of tiles. Since $f_{\mathrm{L}} = a_{k-1}f_\alpha + a_k\sum_{i=0}^{2} f_{\beta_i} + a_{k+1}f_\gamma$, we prove that each L tile belongs to α, β_i $(i = 0,1,2)$ or γ domain, and β_3 domains never appear in the hexagonal metallic-mean tiling with $k \neq 1$. As for the golden-mean tiling with $k = 1$, α and β_0 domains do not appear, but β_3 domains appear due to the existence of the F vertices. The frequencies of the β_1, β_2, and γ domains are given by Eqs. (21), (22), and (23), and the frequency of the β_3 domains is given as

$$f_{\beta_3} = \frac{r_1 f_{\mathrm{F}}}{\tau_1^2} = \frac{5+3\tau_1}{31\tau_1^9}. \tag{24}$$

We show in Suppl. Fig. 5 the frequency of the L tiles which belong to each domain. When k is small, the β_1, β_2, and γ domains are majority in the tilings. On the other hand, when the system approaches the honeycomb lattice, α and β_0 domains become dominant in the system. This originates from the fact that, in the large k case, the vertices are almost composed of the C_1 and C_0 vertices, and thereby α and β_0 domains, which are generated by applying the deflation operation to the above vertices, become dominant.

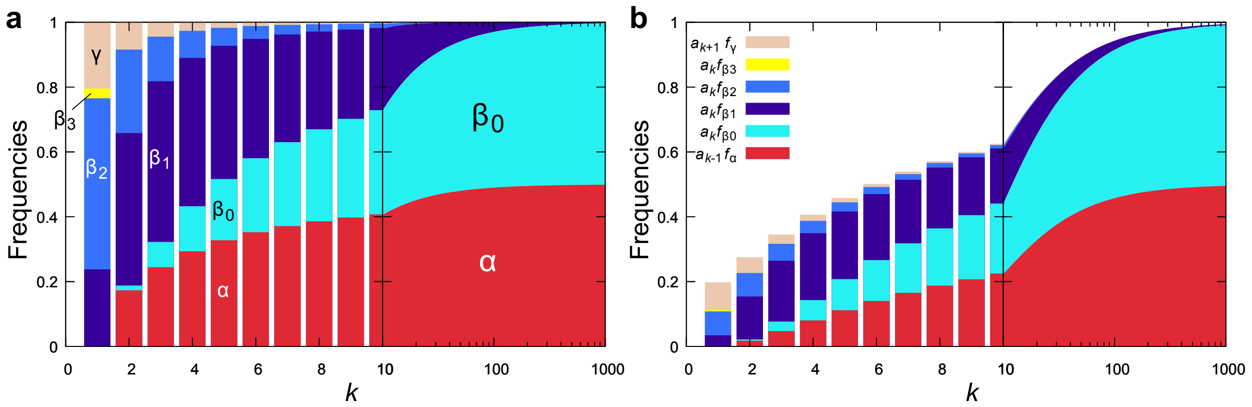

Supplementary Fig. 5. **Frequencies of the honeycomb domains.** **a** Frequencies of α, β_i $(i = 0, 1, 2, 3)$, and γ domains for the total number of the domains are shown as the cumulative bar chart. **b** Frequency of the L tiles belonging to each domain are shown as the cumulative bar chart. Source data are provided as a Source Data file.

Supplementary Note 4 Domain boundaries

Honeycomb domains are separated by the P and S tiles and are distributed like triangular structure, as shown in Suppl. Fig. 2. This may allow us to regard the hexagonal metallic-mean tilings as the honeycomb lattice modulated by the one-dimensional domain boundaries along three directions with the angles $\theta = 0$ and $\pm\pi/3$. Here, we clarify how the domain boundaries are distributed in the metallic-mean tilings with $k \neq 1$.

Suppl. Fig. 6a,c shows the domain boundaries along the horizontal direction as colored tiles for $k = 2$ and $k = 3$. Each domain boundary is composed of the P and S tiles, and adjacent P tiles always share their shorter edges. We find that each P tile is adjacent to its reflected P tile ($\bar{\mathrm{P}}$ tile) or an S tile. Therefore, the zigzag chains of P tiles appear in the domain boundary. We note that three domain boundaries with distinct directions do not share any P tiles, but always cross at a certain S tile.

Now, we discuss how the domain boundaries along a certain direction are arranged in the metallic-mean tilings. We find in Suppl. Fig. 6a,c that the spaces between the domain boundaries are classified by two groups $\mathcal{S}_\mathrm{S}$ and $\mathcal{S}_\mathrm{L}$. In the smaller space $\mathcal{S}_\mathrm{S}$, there exist the α and β honeycomb domains, and in the other space $\mathcal{S}_\mathrm{L}$, β and γ domains appear. To clarify the distributions of these spaces, we use the substitution rule of the tiles. Suppl. Fig. 6b and 6d shows the deflations of the tilings shown in Suppl. Fig. 6a,c, respectively. When the deflation operation is applied to the tiling, k and $k+1$ domain boundaries are equally-spaced generated in the original $\mathcal{S}_\mathrm{S}$ and $\mathcal{S}_\mathrm{L}$ spaces, respectively. Namely, $k-1$ and k $\mathcal{S}_\mathrm{S}$ are generated. On the other hand, under one deflation operation, one $\mathcal{S}_\mathrm{L}$ is generated at each domain boundary. Therefore, the numbers of $\mathcal{S}_\mathrm{S}$ and $\mathcal{S}_\mathrm{L}$ at iteration n ($N_{\mathcal{S}_\mathrm{S}}$ and $N_{\mathcal{S}_\mathrm{L}}$) satisfy

$$\begin{pmatrix} N_{\mathcal{S}_\mathrm{S}}^{(n+1)} \\ N_{\mathcal{S}_\mathrm{L}}^{(n+1)} \end{pmatrix} = \begin{pmatrix} k-1 & k \\ 1 & 1 \end{pmatrix} \begin{pmatrix} N_{\mathcal{S}_\mathrm{S}}^{(n)} \\ N_{\mathcal{S}_\mathrm{L}}^{(n)} \end{pmatrix}, \tag{25}$$

where the maximum eigenvalue is given by τ_k and the corresponding eigenvector $(k\tau_k, 1+\tau_k)^T$. This means that the self-similarity inherent in the metallic ratio τ_k appears in the tiles, honeycomb domains, and spaces between adjacent domain boundaries.

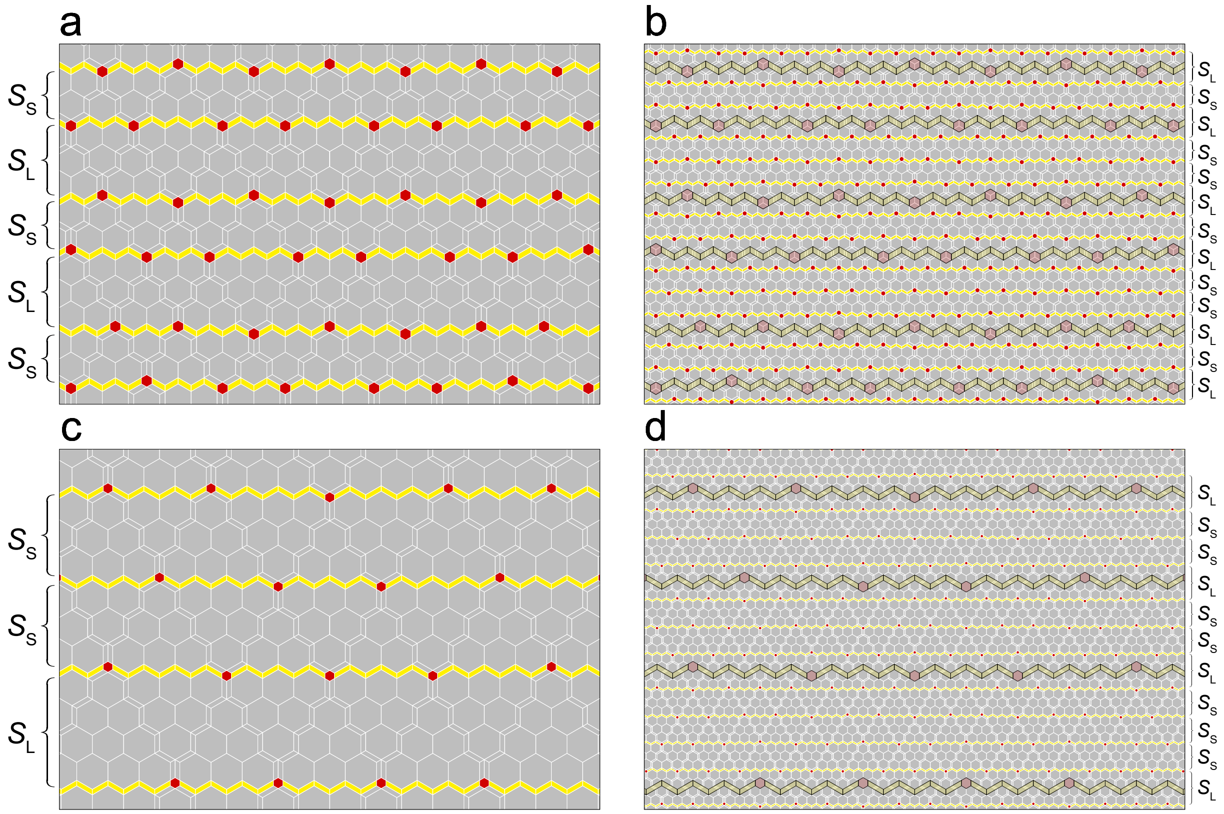

Supplementary Fig. 6. **Domain boundaries in the hexagonal metallic-mean tilings. a (c)** The colored tiles represent the domain boundaries along the horizontal direction for the hexagonal silver-mean (bronze-mean) tiling with $k = 2$ ($k = 3$). **b (d)** The tilings are obtained by applying the deflation operation to the ones shown in **a** (**c**). The transparent tiles represent the original domain boundaries along the horizontal direction.

Supplementary Note 5 Higher-dimensional representation

Perpendicular space

In this section, we consider the perpendicular space, introducing the six-dimensional representations of the vertices. First, we describe the vertex site in the two-dimensional physical space $\mathcal{S}$ by six vectors $\mathbf{e}_m$ and six integer indices $\vec{\mathrm{n}} = (n_0, n_1, n_2, n_3, n_4, n_5)^T$, as

$$\mathbf{r} = (x, y) = \sum_{m=0}^{5} n_m \mathbf{e}_m, \tag{26}$$

with

$$\mathbf{e}_m = \begin{cases} \Big(\ell\cos(m\phi+\theta), \ell\sin(m\phi+\theta)\Big) & m = 0,1,2 \\ \Big(s\cos(m\phi+\theta), s\sin(m\phi+\theta)\Big) & m = 3,4,5 \end{cases}, \tag{27}$$

where $\phi = 2\pi/3$ and θ is constant. ℓ and s are the lengths for longer and shorter edges of the tiles. The vectors $\mathbf{e}_m$ are schematically shown in Suppl. Fig. 7.

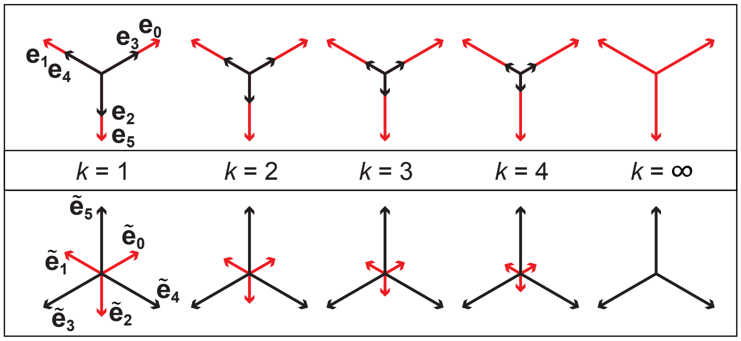

Supplementary Fig. 7. **Basis vectors in real and perpendicular spaces.**

In Suppl. Eq. (26), the vertex site $\mathbf{r}$ can be regarded as the projection from a six-dimensional lattice point, where the vectors $\mathbf{e}_m$ are the projections from the six-dimensional basis vectors. Thereby one can define the projections onto the other four-dimensional space (perpendicular space). For a unified understanding of the projection, it is convenient to introduce the six-dimensional space $\mathcal{S}^h$ including the physical and perpendicular spaces.

Then, $\vec{\mathrm{n}}$ is mapped to the six-dimensional lattice point $\vec{\mathrm{r}}^h$ in $\mathcal{S}^h$ as,

$$\vec{\mathrm{r}}^h = M\vec{\mathrm{n}}, \tag{28}$$

$$M = \begin{pmatrix} \ell\cos\theta & \ell\cos(\phi+\theta) & \ell\cos(2\phi+\theta) & s\cos\theta & s\cos(\phi+\theta) & s\cos(2\phi+\theta) \\ \ell\sin\theta & \ell\sin(\phi+\theta) & \ell\sin(2\phi+\theta) & s\sin\theta & s\sin(\phi+\theta) & s\sin(2\phi+\theta) \\ \tau_k^{-1}\cos\theta & \tau_k^{-1}\cos(\phi+\theta) & \tau_k^{-1}\cos(2\phi+\theta) & -\cos\theta & -\cos(\phi+\theta) & -\cos(2\phi+\theta) \\ \tau_k^{-1}\sin\theta & \tau_k^{-1}\sin(\phi+\theta) & \tau_k^{-1}\sin(2\phi+\theta) & -\sin\theta & -\sin(\phi+\theta) & -\sin(2\phi+\theta) \\ \sqrt{2}\tau_k^{-1} & \sqrt{2}\tau_k^{-1} & \sqrt{2}\tau_k^{-1} & 0 & 0 & 0 \\ 0 & 0 & 0 & -\sqrt{2} & -\sqrt{2} & -\sqrt{2} \end{pmatrix}, \tag{29}$$

where M is the mapping matrix. Then, one can discuss vertex properties in both physical and perpendicular space. Namely, in the physical space $\mathcal{S}$, the vertex site $\mathbf{r}$ is given by the first two components of the vector as

$$\mathbf{r} = \Big((\vec{\mathrm{r}}^h)_0, (\vec{\mathrm{r}}^h)_1\Big) = \sum_{m=0}^{5} n_m \mathbf{e}_m. \tag{30}$$

The four-dimensional perpendicular space is split into two-dimensional spaces $\tilde{\mathcal{S}}$ and $\mathcal{S}^\perp$, and the corresponding coordinates $\tilde{\mathbf{r}}$ and $\mathbf{r}^\perp$ are given as

$$\tilde{\mathbf{r}} = \Big((\vec{\mathrm{r}}^h)_2, (\vec{\mathrm{r}}^h)_3\Big) = \sum_{m=0}^{5} n_m \tilde{\mathbf{e}}_m, \tag{31}$$

$$\mathbf{r}^\perp = \Big((\vec{\mathrm{r}}^h)_4, (\vec{\mathrm{r}}^h)_5\Big) = \sum_{m=0}^{5} n_m \mathbf{e}_m^\perp, \tag{32}$$

where $\tilde{\mathbf{e}}_m = (M_{2m}, M_{3m})$ and $\mathbf{e}_m^\perp = (M_{4m}, M_{5m})$. We find that $\mathbf{r}^\perp = (x^\perp, y^\perp)$ takes four values $x^\perp = 0, \sqrt{2}\tau_k^{-1}$ and $y^\perp = -\sqrt{2}, 0$ in the hexagonal metallic-mean tilings. In each $\mathbf{r}^\perp$ plane, the $\tilde{\mathbf{r}}$ points densely cover a certain window. We find that the window in planes $\mathbf{r}^\perp = (0,0), (\sqrt{2}\tau_k^{-1}, -\sqrt{2})$ $[(0,-\sqrt{2}), (\sqrt{2}\tau_k^{-1}, 0)]$ has a hexagonal (triangular) structure. Suppl. Fig. 8 shows the perpendicular spaces for the hexagonal metallic-mean tilings with $k = 1, 2, 3, 4$, and ∞. The characteristic lengths λ_1, λ_2 and λ_3 will be obtained in Suppl. Note 6. We find that eight types of vertices are mapped into specific regions. This implies that the perpendicular spaces reflect the local environments for the lattice sites. Namely, the areas of each vertex region in perpendicular spaces are proportional to its frequency in the physical space. The region of the F (C_0) vertices appears only in the case of $k = 1$ ($k \neq 1$). This means the absence of C_0 (F) vertices in the hexagonal metallic-mean tilings with $k = 1$ ($k \neq 1$),

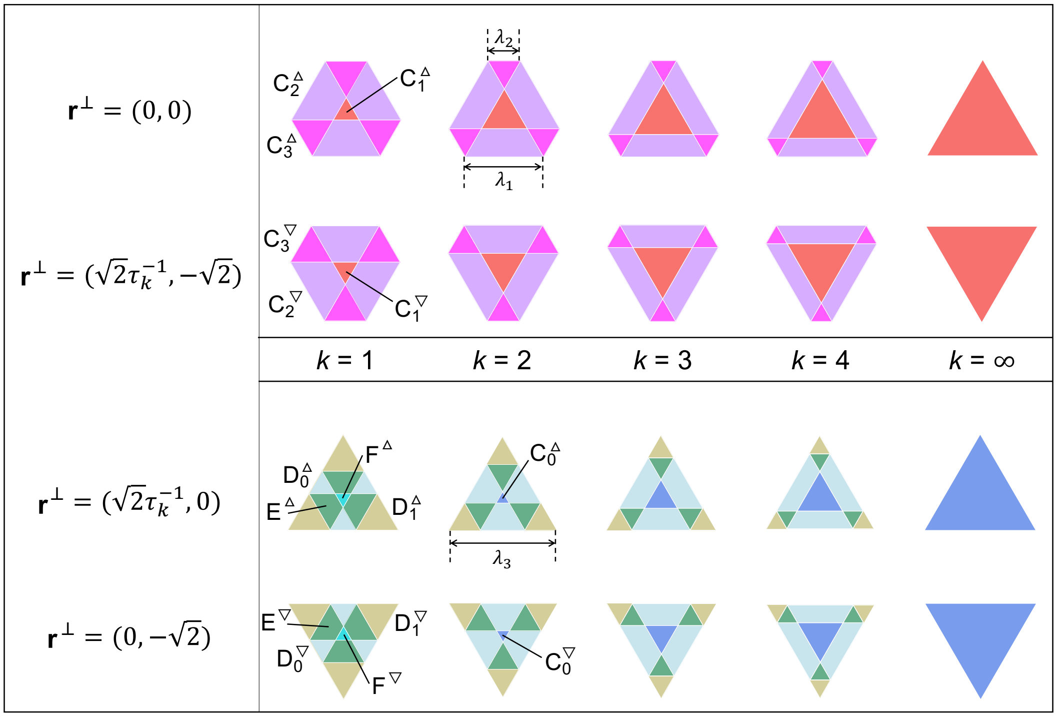

Supplementary Fig. 8. **Perpendicular spaces [$\mathbf{r}^\perp = (0,0), (\sqrt{2}\tau_k^{-1}, -\sqrt{2}), (\sqrt{2}\tau_k^{-1}, 0)$, and $(0, -\sqrt{2})$] of the hexagonal metallic-mean tilings for $k = 1, 2, 3, 4$, and ∞.** Each area bounded by the solid lines is the region of one of eight types of vertices with $\triangle$ and $\triangledown$. λ_1 and λ_2 (λ_3) are the characteristic lengths of the windows with $\mathbf{r}^\perp = (0,0)$ and $(\sqrt{2}\tau_k^{-1}, -\sqrt{2})$ [$(\sqrt{2}\tau_k^{-1}, 0)$ and $(0, -\sqrt{2})$].

which is consistent with the results discussed in the main text and Suppl. Note 2. The areas of the C_0, C_1 vertices (the others) monotonically increase (decrease) with increasing k. The planes $\mathbf{r}^\perp = (0,0)$ and $(\sqrt{2}\tau_k^{-1}, -\sqrt{2})$ [$(0, -\sqrt{2})$ and $(\sqrt{2}\tau_k^{-1}, 0)$] are fully occupied only by the C_0 and C_1 vertices in the limit $k \to \infty$ since $f_{C_0}, f_{C_1} \to 1/2$. We also find that the vertices in the A (B) sublattice are mapped to the planes with $(0,0)$ and $(\sqrt{2}\tau_k^{-1}, -\sqrt{2})$ [$(0, -\sqrt{2})$ and $(\sqrt{2}\tau_k^{-1}, 0)$]. This can be explained by the following. The sublattice index for each vertex is uniquely determined, as discussed above. Since upon moving from one site to its neighbor only one of the n_m's changes by ± 1, the site with an even (odd) number $(\tau_k x^\perp + y^\perp)/\sqrt{2}$ corresponds to the A (B) sublattice. Correspondingly, the areas for both sublattices are different from each other, meaning the existence of the sublattice imbalance in the system.

We wish to note that the perpendicular space analysis clarifies whether each vertex belongs to the honeycomb domain with $\triangle$ or $\triangledown$. As seen in Suppl. Fig. 9, the vertices in the space $\mathbf{r}^\perp = (\sqrt{2}\tau_k^{-1}, -\sqrt{2})$ and $(0, -\sqrt{2})$ [(0,0) and $(\sqrt{2}\tau_k^{-1}, 0)$] belong to the honeycomb domain with $\triangle$ ($\triangledown$). This can be explained by the following. Each honeycomb domain is composed of only L tiles. When one moves within the domain, the longer length appears in the physical space, changing $x^\perp$. Therefore, we find that vertices in a certain honeycomb domain take the common value of $y^\perp$. Furthermore, any honeycomb domains with $\alpha(= \triangle$ or $\triangledown)$ are adjacent to the honeycomb domains with $\bar{\alpha}$ across the single boundary composed of the zig-zag P tiles, as discussed in the main text. When one moves from one honeycomb domain to its neighboring honeycomb domain, one shorter length appears in the physical space, changing $y^\perp$ by $\pm\sqrt{2}$. Therefore, we find that vertices of the adjacent domains take the different values of $y^\perp$ each other, and understand that vertices, which are mapped to the perpendicular spaces $\mathbf{r}^\perp = (\sqrt{2}\tau_k^{-1}, -\sqrt{2})$ and $(0, -\sqrt{2})$ [(0,0) and $(\sqrt{2}\tau_k^{-1}, 0)$], belong to the honeycomb domains with $\triangle$ ($\triangledown$). By these reasons, the perpendicular analysis clarifies not only the sublattice structure but also the honeycomb domain structure. One can define the vertex belonging to the honeycomb domain with $\alpha(= \triangle, \triangledown)$ as X^α, where $X(= \mathrm{C}_0, \mathrm{C}_1, \cdots, \mathrm{F})$. Namely, the frequencies for $X^\triangle$ and $X^\triangledown$ are identical since the corresponding areas are identical in the perpendicular space.

Cut-and-project scheme

In the six-dimensional representations, we have introduced two lengths ℓ and s, and the metallic mean τ_k. Note that, in Suppl. Eq. (29), the length scale appears only in the physical space and the metallic mean appears only in the perpendicular space. This may mean that the metallic mean is important in the perpendicular space while plays no role in the physical space. This allows us to generate the hexagonal metallic-mean tilings with arbitrary lengths ℓ and s, in contrast to the tilings generated by means of the substitution rule. This is known as the cut-and-project scheme, which should be useful to clarify how relevant the tiling is for the atomic position in the MC and MD simulations discussed in the main text.

The six-dimensional lattice points $\vec{\mathrm{r}}^h$ with indices $\vec{\mathrm{n}}$ relevant for the generalized hexagonal metallic-mean tiling satisfies the condition that their projections onto the perpendicular space are located inside the windows, which are shown in Suppl. Fig. 8. Then, projecting

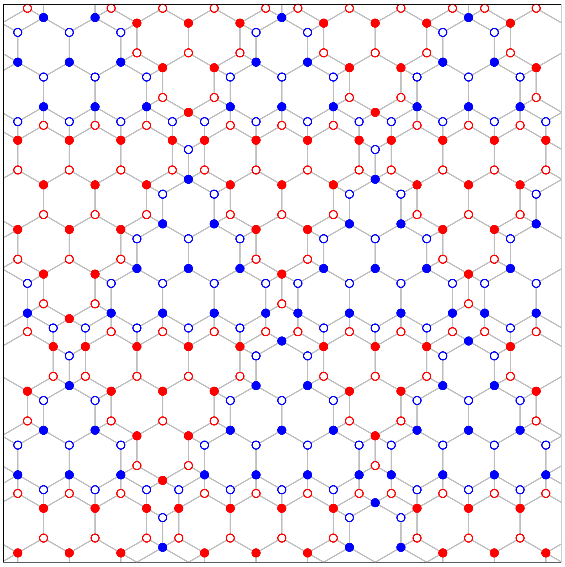

Supplementary Fig. 9. **Hexagonal bronze-mean tiling.** Red open, blue open, red filled, and blue filled circles represent the vertices, which are mapped to the perpendicular space with $\mathbf{r}^{\perp} = (0,0), (\sqrt{2}\tau_k^{-1}, -\sqrt{2}), (\sqrt{2}\tau_k^{-1}, 0)$ and $(0, -\sqrt{2})$, respectively. The blue (red) symbols belong to honeycomb domains with $\triangle$ ($\triangledown$).

these six-dimensional coordinates onto the physical space, we can obtain the generalized hexagonal tilings. Golden-mean, silver-mean, and bronze-mean tilings with $\ell/s = 1/2$ and $\ell/s = 2$ generated by means of the cut-and-project scheme are shown in Suppl. Fig. 10. We wish to note that the self-similarity in the tilings is inherent in the case $\ell/s = \tau_k$ and the substitution rule cannot be defined in generic case with arbitrary ℓ and s.

Reciprocal vectors

Each vertex site in the tilings is described by the six integer indices $\vec{\mathrm{n}}$ and that mapped to the six-dimensional space $\mathcal{S}^h$ is represented as

$$\vec{\mathrm{r}}^h = M\vec{\mathrm{n}} = \sum_i n_i \vec{\mathrm{e}}_i^h, \tag{33}$$

where $\vec{\mathrm{e}}_i^h$ $(i = 0, 1, \cdots, 5)$ are the six kinds of six-dimensional basis vectors with $(\vec{\mathrm{e}}_i^h)_j [= M_{ij}]$. The six kinds of six-dimensional reciprocal vectors $\vec{\mathrm{q}}_i^h$ are obtained by imposing the condition

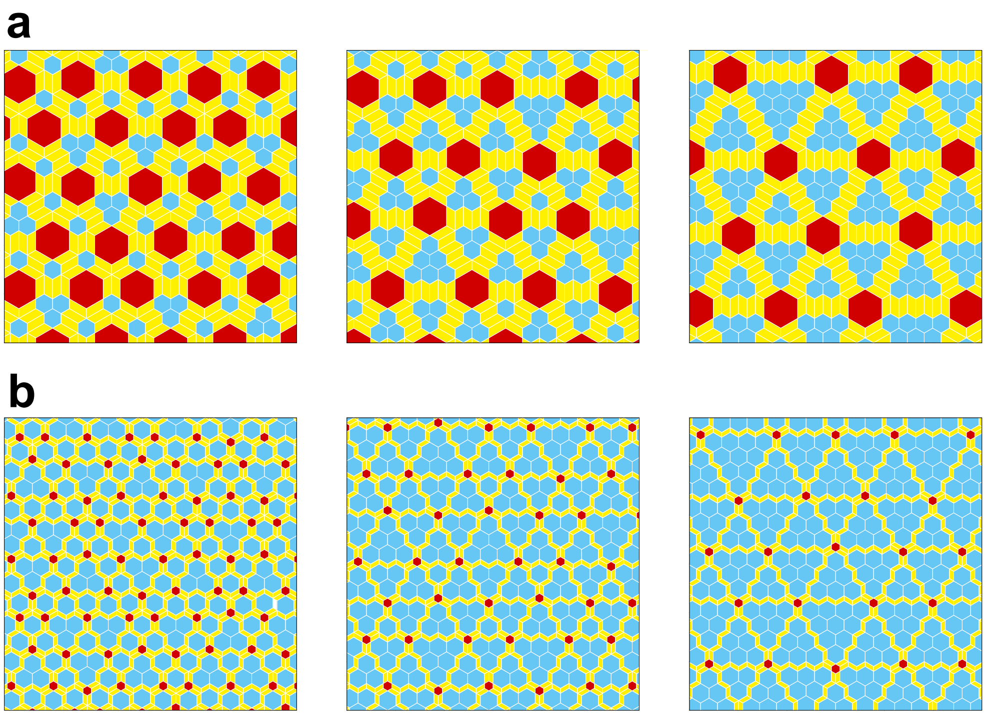

Supplementary Fig. 10. **Hexagonal golden-, silver- and bronze-mean tilings.** These are generated by the cut-and-project scheme with **a** $\ell/s = 1/2$ and **b** $\ell/s = 2$.

$\vec{\mathrm{e}}_i^h \cdot \vec{\mathrm{q}}_j^h = 2\pi\delta_{ij}$ with δ_{ij} is Kronecker delta. These are explicitly given as,

$$\vec{\mathrm{q}}_m^h = C \begin{pmatrix} \cos(m\phi+\theta) \\ \sin(m\phi+\theta) \\ s\cos(m\phi+\theta) \\ s\sin(m\phi+\theta) \\ (\ell\tau_k + s)/(2\sqrt{2}) \\ 0 \end{pmatrix} \qquad \text{for} \quad m = 0, 1, 2, \tag{34}$$

$$\vec{\mathrm{q}}_m^h = C \begin{pmatrix} \tau_k^{-1}\cos(m\phi+\theta) \\ \tau_k^{-1}\sin(m\phi+\theta) \\ -\ell\cos(m\phi+\theta) \\ -\ell\sin(m\phi+\theta) \\ 0 \\ -(\ell + s\tau_k^{-1})/(2\sqrt{2}) \end{pmatrix} \qquad \text{for} \quad m = 3, 4, 5, \tag{35}$$

where $C = 4\pi/[3(\ell + s\tau_k^{-1})]$. The reciprocal vectors projected onto the physical space are given as,

$$\mathbf{q}_m = \Big((\vec{\mathrm{q}}_m^h)_0, (\vec{\mathrm{q}}_m^h)_1\Big). \tag{36}$$

The six reciprocal vectors are composed of three long vectors $\mathbf{q}_0, \mathbf{q}_1, \mathbf{q}_2$ and three short vectors $\mathbf{q}_3, \mathbf{q}_4, \mathbf{q}_5$. The ratio of their lengths is given by τ_k. The longer length $|\mathbf{q}_0| = |\mathbf{q}_1| = |\mathbf{q}_2| = 4\pi/[3(\ell + s\tau_k^{-1})]$ monotonically increases with increasing k and approaches the constant $4\pi/(3\ell)$ in larger k. On the other hand, the shorter length $|\mathbf{q}_3| = |\mathbf{q}_4| = |\mathbf{q}_5| = 4\pi/[3(\ell\tau_k + s)]$ monotonically decreases and vanishes in the limit $k \to \infty$. This is consistent with the fact that, in the limit $k \to \infty$, three of the reciprocal vectors are reduced to those for the honeycomb lattice and the metallic-mean tiling can be regarded as the aperiodic approximant of the honeycomb lattice. We wish to note that the net number of the reciprocal vectors is two due to satisfying $\mathbf{q}_0 = -(\mathbf{q}_1 + \mathbf{q}_2)$ in the limit $k \to \infty$. This is consistent with the fact that the honeycomb lattice is periodic.

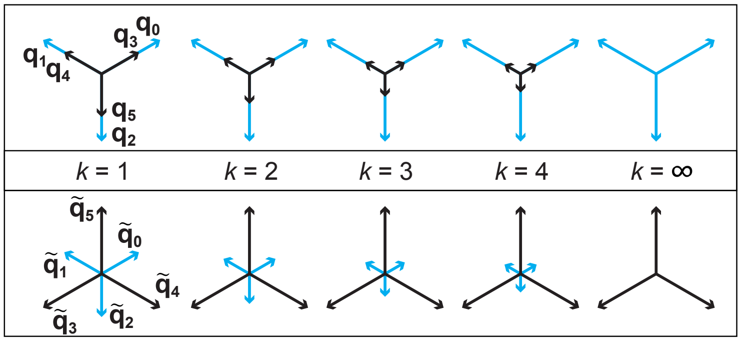

Supplementary Fig. 11. **Reciprocal lattice basis vectors in the real and perpendicular spaces.**

Supplementary Note 6 Phason flips

We examine phason flips in the hexagonal metallic-mean tilings. As seen in Suppl. Note 5, the vertices in the metallic-mean tilings can be mapped inside of the windows in the perpendicular space. When the windows in the perpendicular space slightly slide, positions for some vertices become located outside of the windows and those for some vertices become located inside. This slightly changes the vertex structure in the physical space, that is, some vertices can be regarded to move, so called, phason flips. To discuss how the phason flips occur in the metallic-mean tiling, we show the bronze-mean tiling and the tiling with the slightly shifted windows in Suppl. Fig. 12a,b. For clarify, the difference between these two tilings is shown in color. We find the phason flip along a certain domain boundary. Furthermore, we find that this change can be described by three kinds of local flips. Note that a single local flip never appears due to the matching rule of the tilings. One of local flips is the position change around the C_2 vertex sharing one L tile and two P tiles, as shown in Suppl. Fig. 12c, where the C_2 vertex and the D_0, D_1 and E vertices connected to it by the longer edges change their positions. The local flip around the C_3 vertex also occurs at the same time, as shown in Suppl. Fig. 12d, where the C_3 vertex and the D_1 vertices connected to it by the shorter edges change their positions. The other flip appears at the intersection of two domain boundaries, as shown in Suppl. Fig. 12e. In the case, some D_0, D_1 and E vertices change their positions. When one focuses on the E vertex, the change in the physical space is characterized by $\mathbf{e}_2 - \mathbf{e}_0$, as shown in Suppl. Fig. 13a. The corresponding move in the perpendicular space appears between the corners of the triangular window for E vertices, which is characterized by $\tilde{\mathbf{e}}_2 - \tilde{\mathbf{e}}_0$ schematically shown in Suppl. Fig. 13b. Therefore, an edge length of the window of E vertex in the perpendicular space (if $k = 1$, a longer edge length of the trapezoid) is $|\tilde{\mathbf{e}}_2 - \tilde{\mathbf{e}}_0| = \sqrt{3}\tau_k^{-1}$. We immediately obtain the characteristic lengths of the windows in the perpendicular space as,

$$\lambda_1 = \sqrt{3}, \tag{37}$$

$$\lambda_2 = \sqrt{3}\tau_k^{-1}, \tag{38}$$

$$\lambda_3 = \sqrt{3}(1 + \tau_k^{-1}). \tag{39}$$

We note that the above phason flip also changes the areas of the honeycomb domains. This should be observed as thermal fluctuations of Monte Carlo and MD simulations [2] (See the

main text for the details).

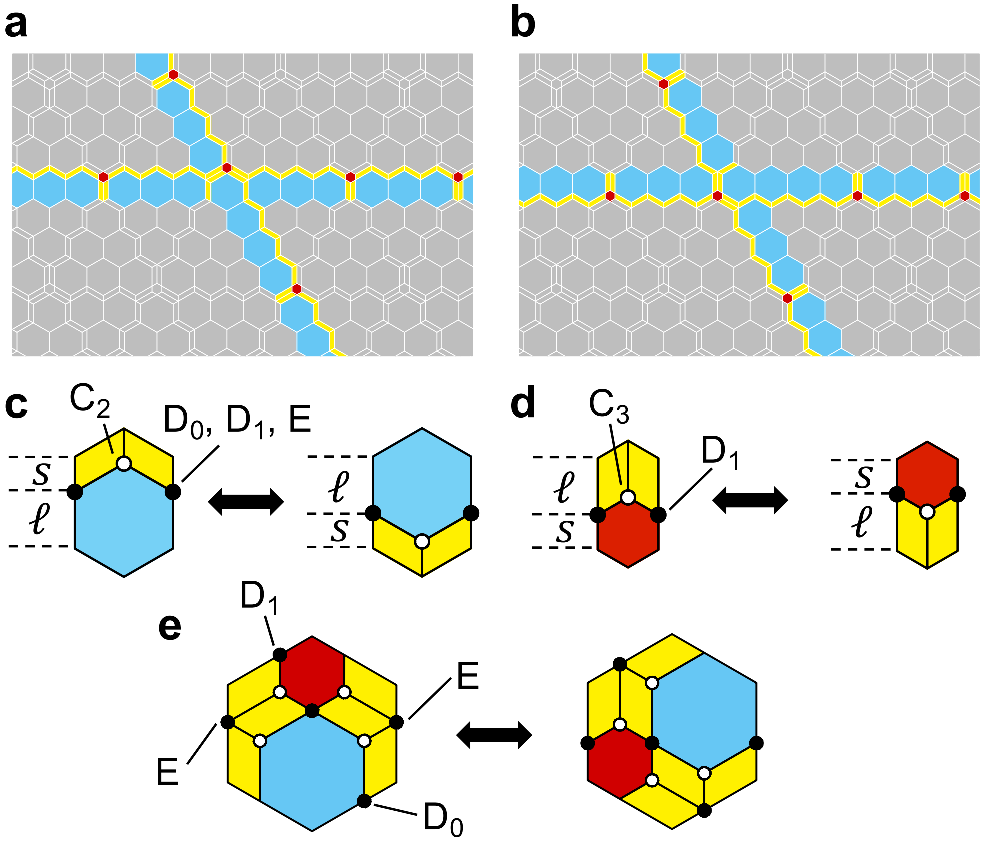

Supplementary Fig. 12. **Schematic pictures for the phason flips in the hexagonal bronze-mean tiling.** **a** and **b** show the bronze-mean tiling and the tiling with the slightly shifted windows, respectively. The difference is explicitly shown in color. **c** (**d**) The local flips around the C_2 (C_3) vertex. **e** The local flip at the intersection of two domain boundaries. In **c**, **d** and **e**, circles represent the vertices which move due to the phason flip.

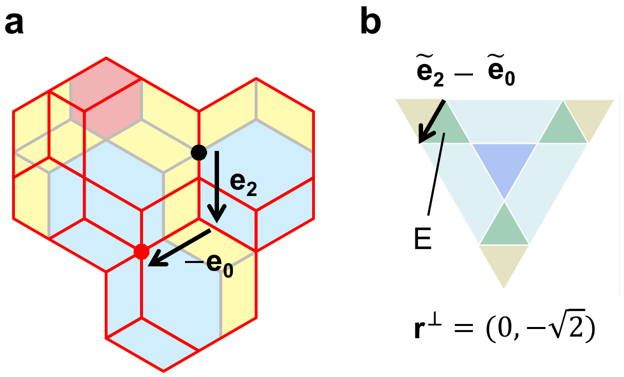

Supplementary Fig. 13. **The position change of the E vertex due to the phason flip.** **a** The black (red) circle indicates the position of the E vertex before (after) the flip, where $\mathbf{e}_i$ is the real space basis vector. **b** shows the corresponding position change in the perpendicular space $\mathbf{r}^{\perp} = (0, -\sqrt{2})$, where $\tilde{\mathbf{e}}_i$ is the perpendicular space basis vector.

Supplementary Note 7 Lattice structure factors

We study the lattice structure factors of the hexagonal metallic-mean tilings in detail. Suppl. Fig. 14a shows the lattice structure factor for each k, where the circular area corresponds to the weight of the peak. It is clearly found that the structure factor have the sixfold rotational symmetry, meaning that the metallic-mean tilings have a hexagonal symmetry.

To clarify how the peak structure is changed with varying k, we show in Suppl. Fig. 14b (c) the crosssection of the lattice structure factors on two mirror axes, which are explicitly shown as the yellow (red) line in Suppl. Fig. 14a. When $k \to \infty$, the peaks are periodically distributed since the system is reduced to the honeycomb lattice. These positions are spanned by $\mathbf{q}_0, \mathbf{q}_1$ and $\mathbf{q}_2$, and the weights of the peaks take two values, 1 and 0.25, as shown in Suppl. Fig. 14b,c. Decreasing k, main peaks remain with larger weights and satellite peaks with smaller weights are induced slightly away from the main peaks. The main peak positions are mainly spanned by $\mathbf{q}_0, \mathbf{q}_1$ and $\mathbf{q}_2$. On the other hand, the other peak positions are represented by six reciprocal vectors as

$$\mathbf{Q} = \sum_{m=0}^{5} \tilde{n}_m \mathbf{q}_m, \tag{40}$$

where $\tilde{n}_m$ is an integer. These are consistent with the fact that the metallic-mean tilings are quasiperiodic. Furthermore, the existence of the satellite peaks originates from the incommensurate modulated structure formed by the domain boundary composed of P tiles in the real space. When $k \leq 4$, the main peaks with large weights are represented by six reciprocal vectors, which are shown as the blue circles in Suppl. Fig. 14b,c. This means that the vertex structures for small k are no longer described by the modulated honeycomb lattice.

Here, we calculate the lattice structure factors. The density of the vertex sites in the tiling is given by $\rho(\mathbf{r}) = \sum_j \delta(\mathbf{r}_j - \mathbf{r})$, where δ is the delta function and $\mathbf{r}_i$ represents the position of the ith vertex site. The summation is over all vertex sites in the tiling. The lattice structure factors are give by $|\rho(\mathbf{q})|^2$, where

$$\rho(\mathbf{q}) = \int \rho(\mathbf{r}) \exp(-i\mathbf{r} \cdot \mathbf{q}) d\mathbf{r} = \sum_j \exp(-i\mathbf{r}_j \cdot \mathbf{q}). \tag{41}$$

It is convenient to make use of the higher-dimensional representation discussed in Suppl. Note 5. Since the six-dimensional basis and reciprocal vectors $\vec{\mathrm{e}}_m^h$ and $\vec{\mathrm{q}}_m^h$ satisfy the orthogonal re-

lation $\vec{e}^h_m \cdot \vec{q}^h_n = 2\pi\delta_{mn}$, we obtain

$$\exp(\mathrm{i}\vec{e}^h_m \cdot \vec{q}^h_n) = \exp(\mathrm{i}\mathbf{e}_m \cdot \mathbf{q}_n)\exp(\mathrm{i}\tilde{\mathbf{e}}_m \cdot \tilde{\mathbf{q}}_n)\exp(\mathrm{i}\mathbf{e}^\perp_m \cdot \mathbf{q}^\perp_n) = 1, \tag{42}$$

where $\mathbf{r}_m(\mathbf{q}_m)$, $\tilde{\mathbf{r}}_m(\tilde{\mathbf{q}}_m)$, and $\mathbf{r}^\perp_m(\mathbf{q}^\perp_m)$ are the two-dimensional vectors in the spaces $\mathcal{S}, \tilde{\mathcal{S}}$, and $\mathcal{S}^\perp$, respectively. Therefore, we obtain

$$\rho(\mathbf{q}) = \sum_j \exp(\mathrm{i}\tilde{\mathbf{r}}_j \cdot \tilde{\mathbf{q}})\exp(\mathrm{i}\mathbf{r}^\perp_j \cdot \mathbf{q}^\perp), \tag{43}$$

where the jth vertex site $\mathbf{r}_j$ is mapped to $\tilde{\mathbf{r}}_j$ and $\mathbf{r}^\perp_j$ in the perpendicular spaces $\tilde{\mathcal{S}}$ and $\mathcal{S}^\perp$. Each vertex site is mapped to the point in one of four domains with $\mathbf{r}^\perp = (0,0), (\sqrt{2}\tau_k^{-1}, -\sqrt{2}), (\sqrt{2}\tau_k^{-1}, 0)$, and $(0, -\sqrt{2})$. Therefore, the Suppl. Equation (43) can be divided into four, as

$$\begin{aligned}\rho(\mathbf{q}) &= \mathcal{F}_{00}(\tilde{\mathbf{q}}) + \exp(\mathrm{i}\sqrt{2}\tau_k^{-1}q^\perp_0)\mathcal{F}_{10}(\tilde{\mathbf{q}}) \\ &\quad + \exp(-\mathrm{i}\sqrt{2}q^\perp_1)\mathcal{F}_{01}(\tilde{\mathbf{q}}) + \exp(\mathrm{i}\sqrt{2}\tau_k^{-1}q^\perp_0)\exp(-\mathrm{i}\sqrt{2}q^\perp_1)\mathcal{F}_{11}(\tilde{\mathbf{q}}), \qquad (44)\\ \mathcal{F}_{mn}(\tilde{\mathbf{q}}) &= \sum_{j\in(mn)^\perp} \exp(\mathrm{i}\tilde{\mathbf{r}}_j \cdot \tilde{\mathbf{q}}), \qquad (45)\end{aligned}$$

where $\mathcal{F}_{mn}$ indicates the Fourier transform of the occupation domain in the perpendicular space with $(\tau_k x^\perp, -y^\perp)/\sqrt{2} = (m, n)$, which are explicitly shown as the hexagons and triangles shown in Suppl. Fig. 8. Then, we can evaluate the lattice structure factor $|\rho(\mathbf{q})|^{\mathbf{2}}$.

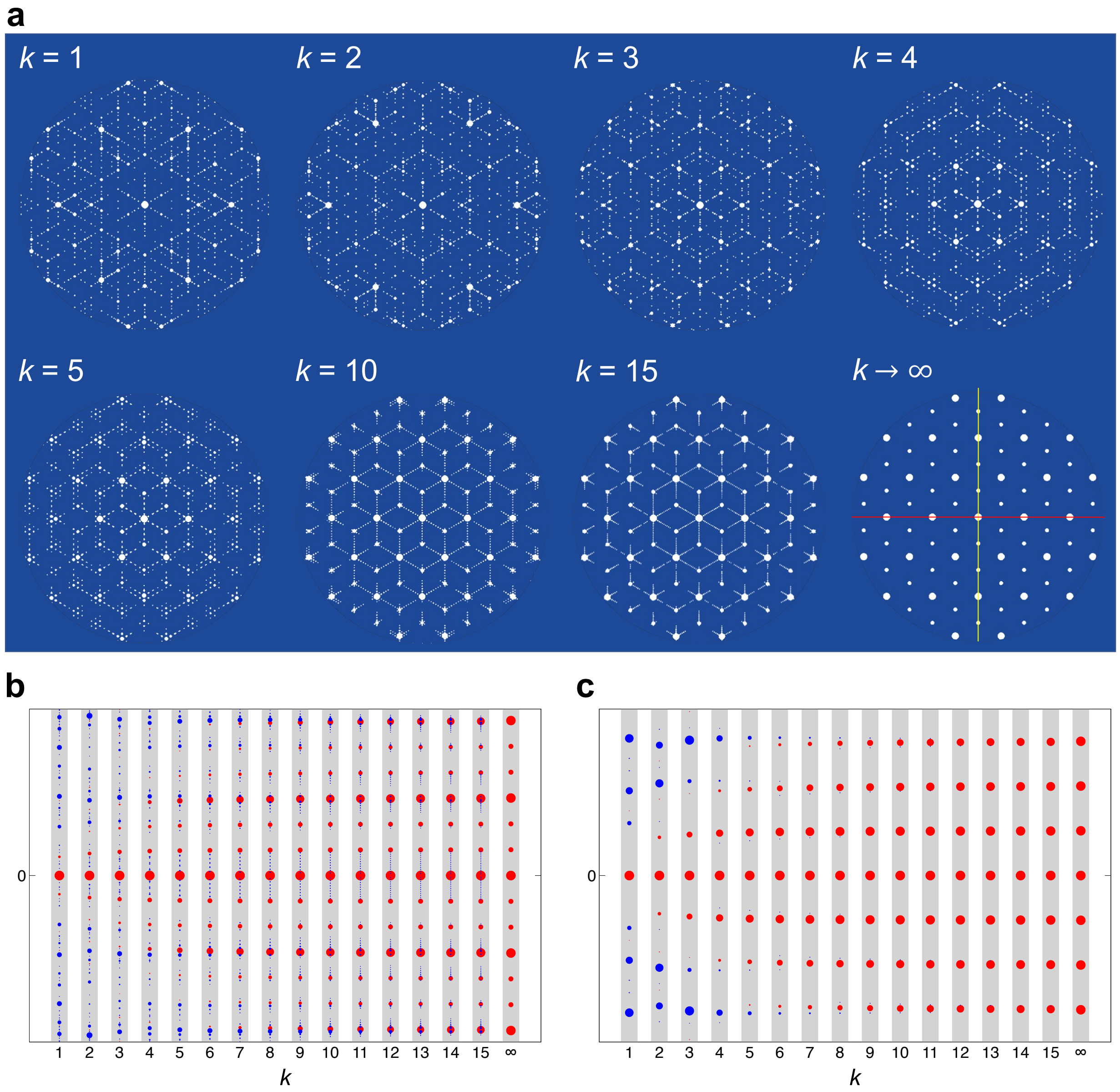

Supplementary Fig. 14. **Lattice structure factors of the hexagonal metallic-mean tilings.** **a** The lattice structure factor for each k. **b** (**c**) The crosssection of the lattice structure factors on the mirror axis shown as the yellow (red) line in **a**. The red circles show peak positions spanned by only $\mathbf{q}_0, \mathbf{q}_1$ and $\mathbf{q}_2$, and the blue circles show the others. Source data are provided as a Source Data file.

Supplementary Note 8 Crystallographic description of the P31m particle system

The particle system exhibits a regular structure with a plane group represented by P31m, as shown in Suppl. Fig. 15. A solid bold line indicates a mirror plane, and a dashed line indicates a glide plane. A solid triangle symbol indicates a three-fold rotation axis. This group is characterized by its 3-fold symmetry. Let A, B, C, D, E, and F be the centers of particles. As a result of this symmetry, we observe that the distances AB, BC, and BD are all equal. To further analyze the system, we make the assumption that there are two types of equilateral polygons present: pentagons and triangles. By considering the equality AB = CF, we can determine the positions of all the particles in the system. Within the unit cell, there are five particles, and their positions can be identified using the Wyckoff Positions 2b and 3c, as shown in the Suppl. Table 1. If the coordinates of particle A in Suppl. Fig. 15a is set to be $(c, 0)$, we find

$$c = \frac{\sqrt{33}-3}{12} = 0.2287, \quad \overline{\mathrm{AB}} = \overline{\mathrm{CF}} = \sqrt{3}c = \frac{\sqrt{11}-\sqrt{3}}{4} = 0.3961. \tag{46}$$

It is important to note that while the pentagon (BCFED) in the particle system is equilateral, it is not a regular pentagon, for it is elongated along the mirror plane.

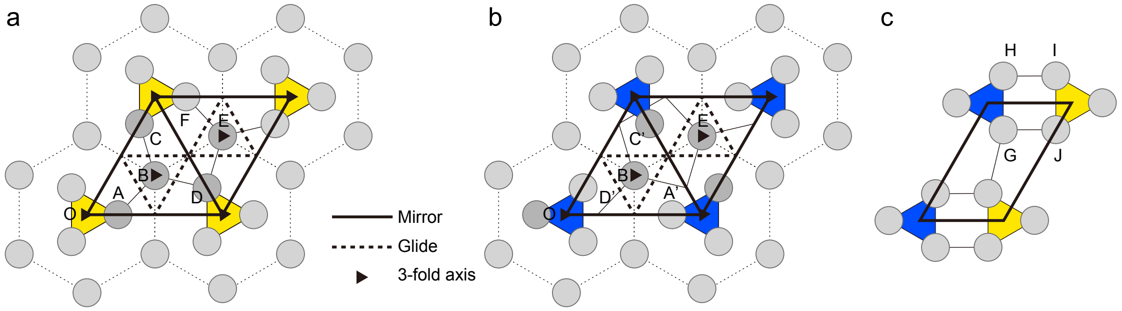

Supplementary Fig. 15. **Plane group P31m for a particle system in distinct domains (a and b), and decoration of a parallelogram (c).**

The basis vectors $\mathbf{a}_1$, $\mathbf{a}_2$ and the corresponding reciprocal basis vectors $\mathbf{b}_1$, $\mathbf{b}_2$ are given by

$$\mathbf{a}_1 = a(1,0), \quad \mathbf{a}_2 = a\left(\frac{1}{2}, \frac{\sqrt{3}}{2}\right), \quad \mathbf{b}_1 = \frac{2\pi}{a}\left(1, -\frac{\sqrt{3}}{3}\right), \quad \mathbf{b}_2 = \frac{2\pi}{a}\left(0, \frac{2\sqrt{3}}{3}\right),$$

Supplementary Table 1. **Wyckoff Position for a particle system.** $c = \frac{\sqrt{33}-3}{12} = 0.2287$.

Wyckoff Position	Site Sym.	Points	Coordinates
2b	3..	B, E	$\left(\frac{1}{2}, \frac{\sqrt{3}}{6}\right), \left(1, \frac{\sqrt{3}}{3}\right)$
3c	..m	A, C, D	$(c, 0), \left(\frac{1}{2}(1-c), \frac{\sqrt{3}}{2}(1-c)\right), \left(1-\frac{1}{2}c, \frac{\sqrt{3}}{2}c\right)$
3c	..m	A', C', D'	$(1-c, 0), \left(\frac{1+c}{2}, \frac{\sqrt{3}}{2}(1-c)\right), \left(\frac{1}{2}c, \frac{\sqrt{3}}{2}c\right)$

where a is the lattice constant (rhombus edge). For the reciprocal lattice vector $\mathbf{G} = h\mathbf{b}_1 + k\mathbf{b}_2$, the structure factor is evaluated as

$$F(h,k) = f \sum_{\alpha=\mathrm{A,B,C,D,E}} \exp(2\pi \mathrm{i} \mathbf{G} \cdot \mathbf{r}_\alpha). \tag{47}$$

The diffraction image calculated by this equation is shown in Suppl. Fig. 16.

Model for the metallic-mean tiling: Suppl. Fig. 15c represents a decoration model for a parallelogram. If we assume that the rectangle GHIJ is a square; *i.e.* $\overline{\mathrm{AB}} = \overline{\mathrm{CF}} = \overline{\mathrm{GH}} = \overline{\mathrm{GJ}}$, then the length ratio of the tile is

$$\frac{s}{\ell} = (1+\sqrt{3})c = 0.6249. \tag{48}$$

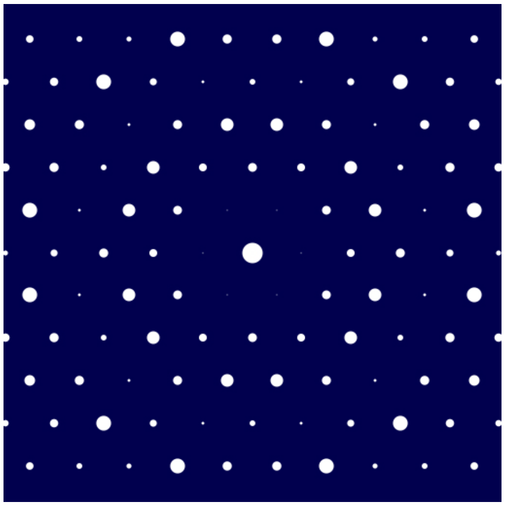

Supplementary Fig. 16. **Diffraction image for P31m perfect crystal ($k \to \infty$).** This is calculated by Suppl. Eq. (48) rotated 90°. The area is proportional to the intensity. Source data are provided as a Source Data file.

Supplementary Note 9 Crystallographic description of the P31m polymer blend system

The same plane group is adopted by an ABC triblock terpolymer/homopolymer blend, where dark gray circles represent polyisoprene (PI), light gray circles represent poly(2-vinylpyridine) (PVP), and the matrix region is polystyrene (PS), as shown in Suppl. Fig. 17. We simply assume that domains of PI and PVP are all circles, and that the centers of polyisoprene domains occupy the same positions as the previous colloidal particle system. Let A, B, $\cdots$, I be the centers of circular domains, and let the distance $\overline{\mathrm{OG}} = d$ for the center positions of PVP. Although it is a crude treatment, we determine $d = \frac{3}{5}$ by minimizing the next (entropic) elastic free-energy function $S(d)$ inside a pentagon (BCFED):

$$S(d) \propto \overline{\mathrm{BI}}^2 + \overline{\mathrm{CI}}^2 + \overline{\mathrm{FI}}^2 + \overline{\mathrm{EI}}^2 + \overline{\mathrm{DI}}^2 = \overline{\mathrm{AG}}^2 + 2\overline{\mathrm{BG}}^2 + 2\overline{\mathrm{DG}}^2. \tag{49}$$

Wyckoff Positions for a polymer blend are shown in Suppl. Table 2.

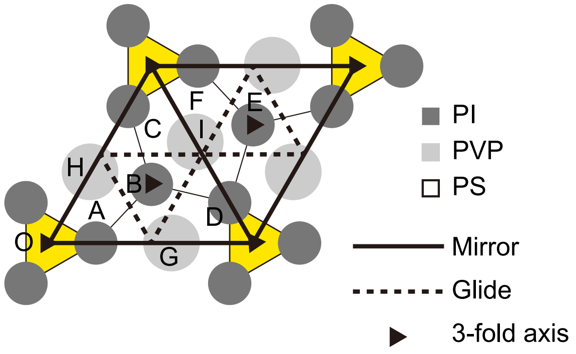

Supplementary Fig. 17. **Plane group P31m for a polymer blend.**

Supplementary Table 2. **Wyckoff Position for a polymer blend.** $c = \frac{\sqrt{33}-3}{12} = 0.2287$ and $d = \frac{3}{5}$.

Polymer	Wyckoff Position	Site Sym.	Points	Coordinates
PI	2b	3..	B, E	$\left(\frac{1}{2}, \frac{\sqrt{3}}{6}\right)$, $\left(1, \frac{\sqrt{3}}{3}\right)$
PI	3c	..m	A, C, D	$(c, 0)$, $\left(\frac{1}{2}(1-c), \frac{\sqrt{3}}{2}(1-c)\right)$, $\left(1-\frac{1}{2}c, \frac{\sqrt{3}}{2}c\right)$
PVP	3c	..m	G, H, I	$(d, 0)$, $\left(\frac{1}{2}(1-d), \frac{\sqrt{3}}{2}(1-d)\right)$, $\left(1-\frac{1}{2}d, \frac{\sqrt{3}}{2}d\right)$

Supplementary Note 10 Pentagon tilings

We demonstrate the accommodation of pentagons within both a square and a hexagon. Firstly, we consider a square with two points placed inside (Suppl. Fig. 18a), and secondly, a hexagon with three points inside (Suppl. Fig. 18b). By connecting the points, we form pentagonal tilings. In Suppl. Fig. 18c, we present the equilateral Cairo pentagonal tiling, which serves as the dual of the $3^2.4.3.4$ Archimedean tiling with the P4gm plane group (Suppl. Fig. 18d). The $3^2.4.3.4$ Archimedean tiling is associated with the σ phase found in complex metallic and soft-matter phases. It is recognized as a periodic approximant of dodecagonal quasicrystals. Additionally, Suppl. Fig. 18e illustrates a 3-fold equilateral pentagon-triangle tiling with the P31m plane group discussed in the present paper. Despite their distinct symmetries, these two structures are closely related and are commonly observed in soft-matter systems.

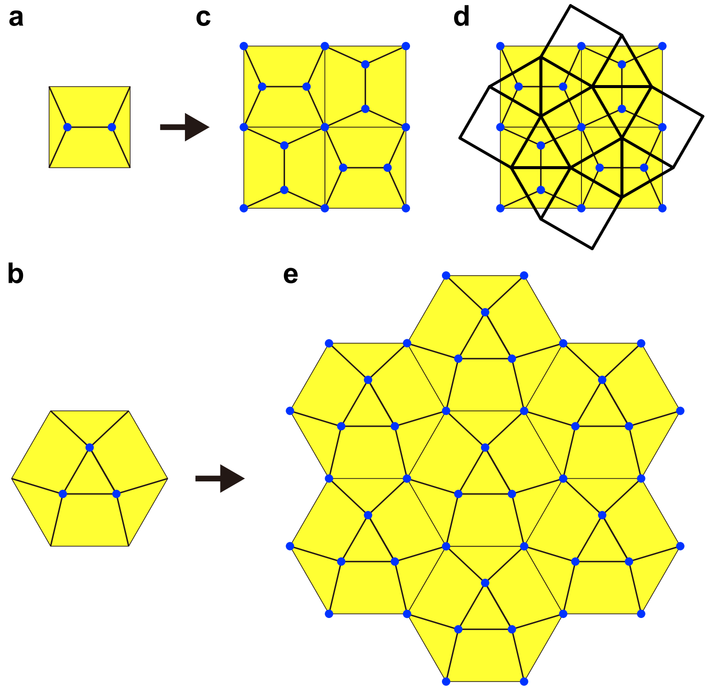

Supplementary Fig. 18. **Equilateral pentagon tilings.** **a** elemental square, **b** elemental hexagon, **c** 4-fold pentagonal Cairo tiling, **d** duality of the Cairo tiling and the $3^2.4.3.4$ Archimedean tiling, and **e** 3-fold pentagon-triangle tiling.

Supplementary Note 11 Colloidal system

Engle used the Lennard-Jones-Gauss potential of the form [2, 3]:

$$V(r) = \frac{1}{r^{12}} - \frac{2}{r^6} - \epsilon \exp\left(-\frac{(r-r_0)^2}{2\sigma^2}\right), \tag{50}$$

with parameters $\sigma^2 = 0.042$, $\epsilon = 1.8$, $r_0 = 1.42$. We have conducted Monte Carlo simulations with NPT ensembles (constant number of particles N, external pressure P, and temperature T) at $T = 0.29$ and $T = 0.28$, $P = 0.0$, and $N = 19740$. Snapshots of modulated structures quenched at $T = 0.12$ are displayed in Suppl. Fig. 19 (a $T = 0.29$ and b $T = 0.28$). The diffraction images in Suppl. Fig. 19 (c $T = 0.29$ and d $T = 0.28$) are obtained by the average of 200 Fourier transforms of quenched samples at $T = 0.12$. In contrast to the diffraction image for the P31m perfect crystal, satellite peaks are observed.

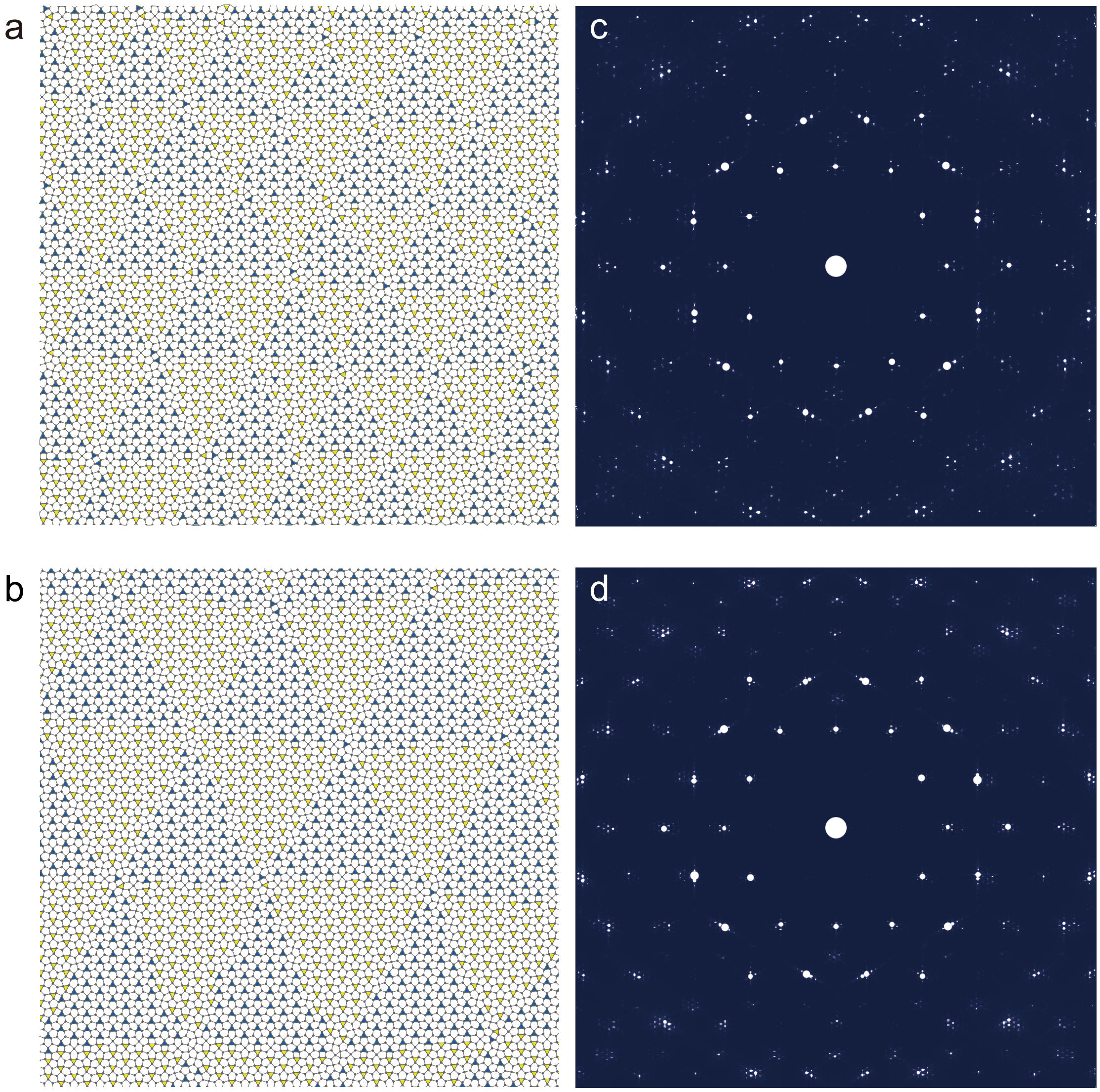

Supplementary Fig. 19. **Colloidal simulations.** Modulated domain structure (**a** $T = 0.29$ and **b** $T = 0.28$) and Diffraction image (**c** $T = 0.29$ and **d** $T = 0.28$). The area is proportional to the intensity. Source data are provided as a Source Data file.

Supplementary Note 12 Atomic decorations

In this section, we consider the decorations on the hexagonal metallic-mean tilings, which should be relevant for the atomic structure of the MC and MD simulations [2]. In a certain parameter regime, MC and MD simulations show the stable solution where the atomic structure is described by large and small hexagons, and parallelograms, as shown in Suppl. Fig. 20. The atomic positions are given in Suppl. Note 8. We find that three atoms are located at the triangle vertices on each vertex. In addition, within each L tile, three atoms are positioned at the triangle vertices at the center, while six atoms are located in the vicinity of each of the six edge centers.

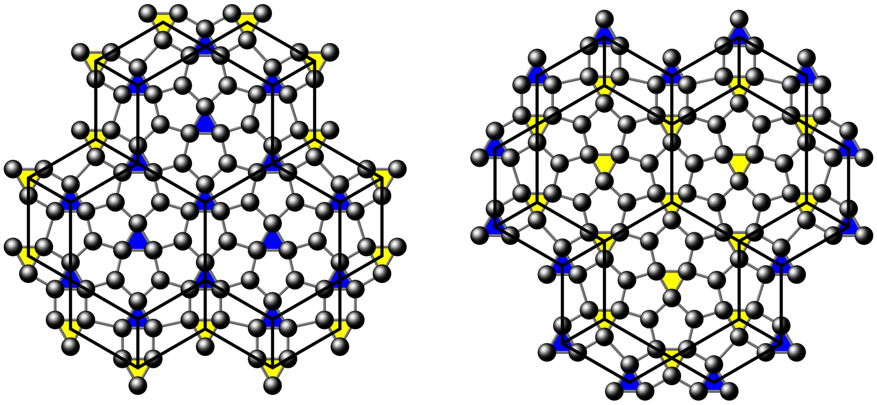

Supplementary Fig. 20. **Atomic structures of the MC and MD simulations.** The circles represent the locations of atoms. Different colored triangles represent different honeycomb domains.

To clarify how relevant the hexagonal metallic-mean tilings proposed in this study are for the atomic structure observed in MD and MC simulations, we first introduce an auxiliary vertex $C_4^{\triangle}$ ($C_4^{\triangledown}$) placed at the center of the $L_{\triangle}$ ($L_{\triangledown}$) tile, as shown in Suppl. Fig. 21a. Since the number of the C_4^{α} ($\alpha = \triangle, \triangledown$) vertices corresponds to that of the L_{α} tiles, its frequency is given as,

$$f_{C_4^{\alpha}} = \frac{1}{4}\frac{\tau_k^2}{\tau_k^2 + 3\tau_k + 1}. \tag{51}$$

When the L_{α} tile is divided into three rhombuses, as shown in Suppl. Fig. 21a, each C_4^{α} vertex connects to the corner vertices in the B sublattice (the solid circles), and it can be regarded to belong to the A sublattice. Since the distance between the C_4^{α} and corner vertices corresponds to the longer length ℓ, the set of the six integer indices for the C_4^{α} vertex

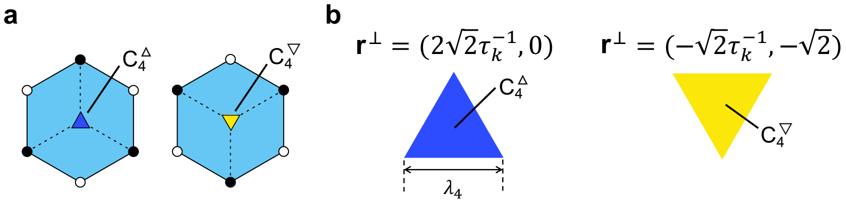

Supplementary Fig. 21. **Auxiliary vertices.** **a** $C_4^\triangle$ and $C_4^\triangledown$ vertices which are defined by dividing $L_\triangle$ and $L_\triangledown$ tiles into three rhombuses. **b** Window of the $C_4^\triangle$ ($C_4^\triangledown$) vertex in the perpendicular space $\mathbf{r}^\perp = (2\sqrt{2}\tau_k^{-1}, 0)$ $[(-\sqrt{2}\tau_k^{-1}, -\sqrt{2})]$. λ_4 is the edge length of the triangles.

is given by

$$\vec{\mathrm{n}}_{C_4^\alpha} = \vec{\mathrm{n}}_{\mathrm{corner}} + \Delta\vec{\mathrm{n}}, \tag{52}$$

where $\vec{\mathrm{n}}_{\mathrm{corner}}$ is the set of the six integer indices for a certain corner vertex in the B sublattice and $\Delta\vec{\mathrm{n}} = (n_0, n_1, n_2, 0, 0, 0)^T$ with integer n_m. This means that the $C_4^\triangle$ and $C_4^\triangledown$ vertices are clearly distinguished in the perpendicular space since $y^\perp$ $[= -\sqrt{2}\vec{\mathrm{n}}_{C_4^\alpha} \cdot (0,0,0,1,1,1)^T]$ for the C_4^α vertices is the same as that for the vertices belonging to the honeycomb domain with α. Namely, the window for the $C_4^\triangle$ ($C_4^\triangledown$) vertex appears in the perpendicular spaces with $\mathbf{r}^\perp = (2\sqrt{2}\tau_k^{-1}, 0)$ $[(-\sqrt{2}\tau_k^{-1}, -\sqrt{2})]$, as shown in Suppl. Fig. 21b. The edge length of the windows is given by $\lambda_4 = \sqrt{3}$, by taking into account the frequency of the vertices. Suppl. Fig. 22 shows the hexagonal bronze-mean tiling with the C_4 vertices, which is obtained by means of the cut-and-projection scheme. We find that the C_4^α auxiliary vertices indeed belong to the honeycomb domain with α. To discuss the spatial structure of the vertices in the perpendicular space in detail, it is useful to consider the corresponding windows in three dimensions $(\tilde{x}, \tilde{y}, \tilde{z} = x^\perp + y^\perp)$, as discussed in the main text. Suppl. Fig. 23 clarifies that these six windows are the crosssections of the trigonal trapezohedron with the edge length $\sqrt{3}(1 + \tau_k^{-1})$. We find that the vertices in the honeycomb domains with $\triangle$ ($\triangledown$) are located in the upper (lower) windows, which are shown as the blue (red) triangles and hexagon. This is consistent with the fact that certain two windows belonging to the same (different) honeycomb domains are bridged by the vectors $\mathbf{e}_0^h, \mathbf{e}_1^h, \mathbf{e}_2^h$ ($\mathbf{e}_3^h, \mathbf{e}_4^h, \mathbf{e}_5^h$). The middle four windows can be regarded as the crosssections of the regular octahedron, which is shown in Fig. 6 in the main text.

Next, we consider the atomic decorations for the hexagonal metallic-mean tilings. One

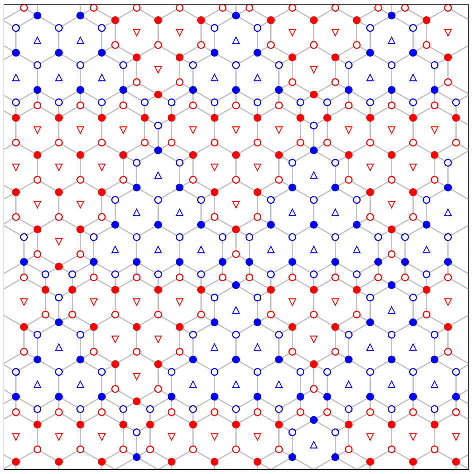

Supplementary Fig. 22. **Hexagonal bronze-mean tiling with the C_4 vertices.** Open (solid) circles at the vertices indicate the sublattice A (B). Open triangles represent the auxeliary C_4 vertices. Blue (red) symbols represent the vertices belonging to the honeycomb domains with $\triangle$ ($\triangledown$).

can divide the atoms into three groups with $\alpha(=\triangle,\triangledown)$, as shown in Suppl. Fig. 24. The group u_1^α is composed of the atoms around the vertex in the hexagonal metallic-mean tiling and the group u_2^α is composed of the atoms around the auxeliary vertex C_4^α (the center of L_α tiles). The other group v^α are located in the L_α tile, but far from the center.

Now, we would like to describe the atomic structures, specifying the sets of the six indices for these atoms. First, we focus on certain atoms in the groups $u_1^\triangle$ and $u_1^\triangledown$ around the vertex $X^\triangle$ and $X^\triangledown$, which are pointed in Suppl. Fig. 24a,b, respectively. We find that the atom with $\alpha=\triangle$ ($\triangledown$) is located in the backward (forward) direction of the vectors $\mathbf{e}_0$ from $X^\triangle$ ($X^\triangledown$). Since the atoms also belong to the honeycomb domain with α, the sets of the six indices $\vec{n}_{\rm atom}$ for these atoms are given as

$$\vec{n}_{\rm atom} = \vec{n}_{X^\alpha} + \vec{n}, \tag{53}$$

$$\vec{n} = (\mp c, 0, 0, 0, 0, 0)^T, \tag{54}$$

where $\vec{n}_{X^\alpha}$ is the set of the six integer indices for the X^α vertex, and the above (under) sign corresponds to $\alpha=\triangle$ ($\triangledown$). In the perpendicular space $\tilde{\mathcal{S}}$, the window of the corresponding atoms appears $\tilde{\mathbf{d}}$ away from the window of X^α, where $\tilde{\mathbf{d}} = \mp c\tilde{\mathbf{e}}_0$. Examining the positions for the atoms of the u_1^α, u_2^α, and v^α groups, we determine the sets of the six indices, which

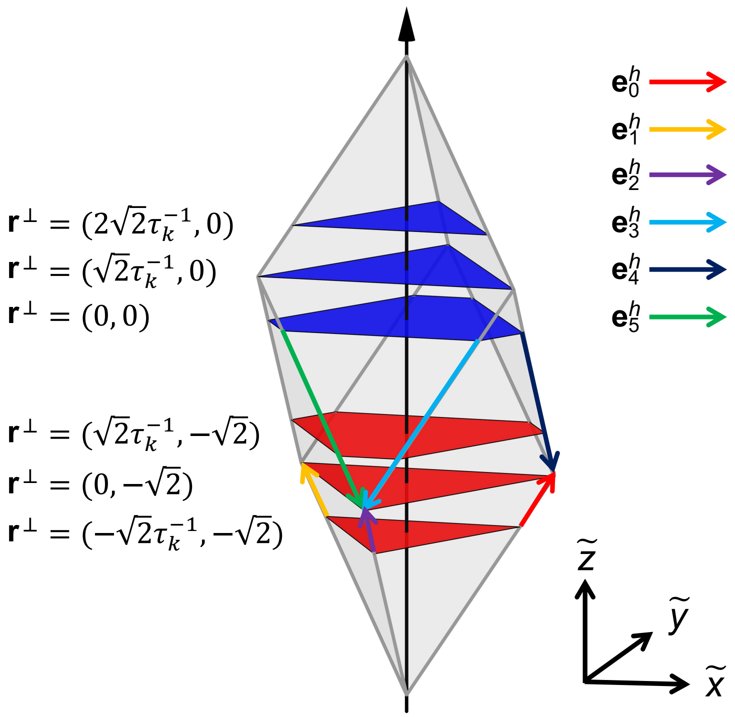

Supplementary Fig. 23. **The perpendicular space [$\mathbf{r}^\perp = (2\sqrt{2}\tau_k^{-1}, 0), (\sqrt{2}\tau_k^{-1}, 0), (0, 0)$, $(\sqrt{2}\tau_k^{-1}, -\sqrt{2}), (0, -\sqrt{2})$, and $(-\sqrt{2}\tau_k^{-1}, -\sqrt{2})$] of the hexagonal bronze-mean tiling with $k = 3$ in three dimensions with $(\tilde{x}, \tilde{y}, \tilde{z} = x^\perp + y^\perp)$.** The colored arrows indicate the projected basis vectors $\mathbf{e}_i^h$ and the blue (red) triangles and hexagon indicate the windows for the honeycomb domains with $\triangle$ ($\triangledown$).

are explicitly shown in Suppl. Table 3. Suppl. Fig. 25a (b) shows the windows for the groups $\mathrm{u}_1^\triangle, \mathrm{u}_2^\triangle$ ($\mathrm{u}_1^\triangledown, \mathrm{u}_2^\triangledown$) in the perpendicular spaces. Suppl. Fig. 26a (b) shows the windows for the group $\mathrm{v}^\triangle$ ($\mathrm{v}^\triangledown$) in the perpendicular space with $\mathbf{r}^\perp = (2\sqrt{2}\tau_k^{-1}, 0)$ [$(-\sqrt{2}\tau_k^{-1}, -\sqrt{2})$]. We generate the hexagonal metallic-mean tilings with the atomic decorations by the cut-and-project scheme, which are schematically shown in Suppl. Fig. 27.

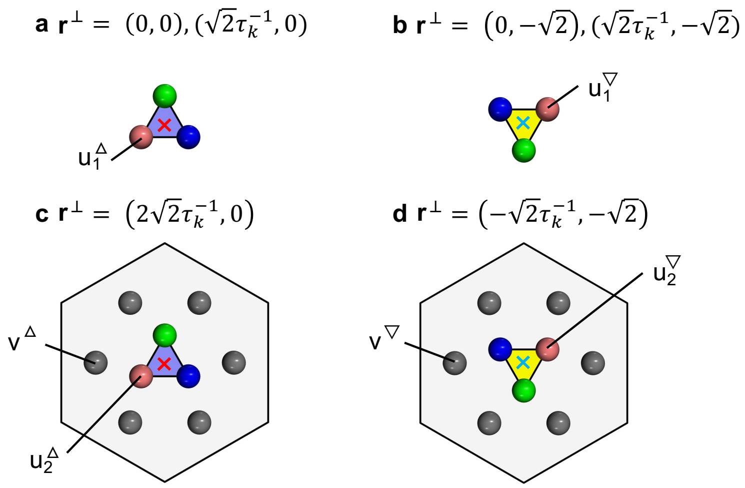

Supplementary Fig. 24. **Positions of atoms in the decorated tiling.** Blue, green, and red circles represent three kinds of the atoms in the u_1 and u_2 groups and gray circles represent the atoms in the v group. **a** (**b**) Red (blue) cross represents the vertex in the honeycomb domain with $\triangle$ ($\bigtriangledown$), which is mapped to the perpendicular space in $\mathbf{r}^\perp = (0,0), (\sqrt{2}\tau_k^{-1}, 0)$ $[(0, -\sqrt{2}), (\sqrt{2}\tau_k^{-1}, -\sqrt{2})]$. **c** (**d**) Atomic decorations of the $L_\triangle$ ($L_\bigtriangledown$) tile. Red (blue) cross represents the auxiliary $C_4^\triangle$ ($C_4^\bigtriangledown$) vertex, which is mapped to the perpendicular space in $\mathbf{r}^\perp = (2\sqrt{2}\tau_k^{-1}, 0)$ $[(-\sqrt{2}\tau_k^{-1}, -\sqrt{2})]$.

Supplementary Table 3. **Occupied plane $\mathbf{r}^{\perp}$ and corresponding window shape in the perpendicular space.** Three sets of six indices for the u_1 and u_2 groups are given, where the above (under) sign corresponds to $\triangle$ ($\triangledown$). Six sets of six indices for the v group are also given. $c = (\sqrt{33} - 3)/12 = 0.2287$.

<table>
<tr><th>Group</th><th>$\mathbf{r}^{\perp}$</th><th>Window</th><th>$3\vec{n}^T$</th></tr>
<tr><td>$\mathrm{u}_1^{\triangle}$</td><td>$(-\sqrt{2}c\tau_k^{-1}, 0), (\sqrt{2}\tau_k^{-1} - \sqrt{2}c\tau_k^{-1}, 0)$</td><td rowspan="2">Hexagon</td><td rowspan="4">$(\mp 3c, 0, 0, 0, 0, 0)$
$(0, \mp 3c, 0, 0, 0, 0)$
$(0, 0, \mp 3c, 0, 0, 0)$</td></tr>
<tr><td>$\mathrm{u}_1^{\triangledown}$</td><td>$(\sqrt{2}c\tau_k^{-1}, -\sqrt{2}), (\sqrt{2}\tau_k^{-1} + \sqrt{2}c\tau_k^{-1}, -\sqrt{2})$</td></tr>
<tr><td>$\mathrm{u}_2^{\triangle}$</td><td>$(2\sqrt{2}\tau_k^{-1} - \sqrt{2}c\tau_k^{-1}, 0)$</td><td rowspan="2">Triangle</td></tr>
<tr><td>$\mathrm{u}_2^{\triangledown}$</td><td>$(-\sqrt{2}\tau_k^{-1} + \sqrt{2}c\tau_k^{-1}, -\sqrt{2})$</td></tr>
<tr><td>$\mathrm{v}^{\triangle}$</td><td>$(2\sqrt{2}\tau_k^{-1}, 0)$</td><td rowspan="2">Triangle</td><td rowspan="2">$(-1, 1, 0, 0, 0, 0)$
$(1, -1, 0, 0, 0, 0)$
$(-1, 0, 1, 0, 0, 0)$
$(1, 0, -1, 0, 0, 0)$
$(0, -1, 1, 0, 0, 0)$
$(0, 1, -1, 0, 0, 0)$</td></tr>
<tr><td>$\mathrm{v}^{\triangledown}$</td><td>$(-\sqrt{2}\tau_k^{-1}, -\sqrt{2})$</td></tr>
</table>

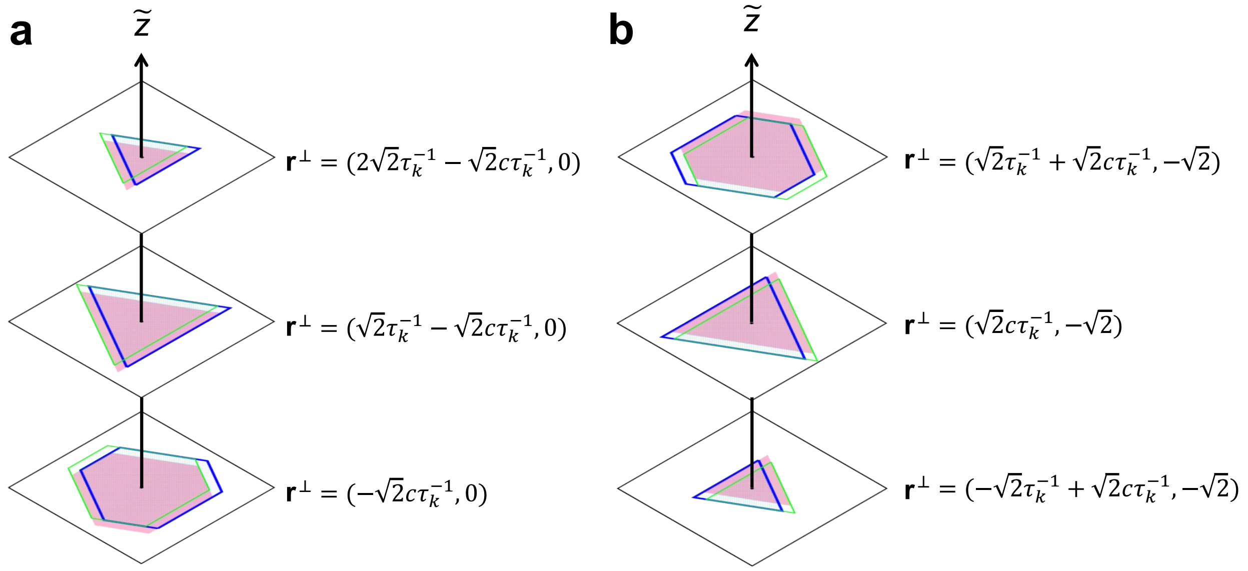

Supplementary Fig. 25. **Windows for the u_1 and u_2 groups in the perpendicular space. a** (**b**) Windows for the $u_1^\triangle, u_2^\triangle$ ($u_1^\triangledown, u_2^\triangledown$) groups in the perpendicular spaces for the hexagonal golden-mean tiling with $k = 1$. Each layer has three distinct windows, whose colors correspond to the colors fo the atom shown in Suppl. Fig. 24.

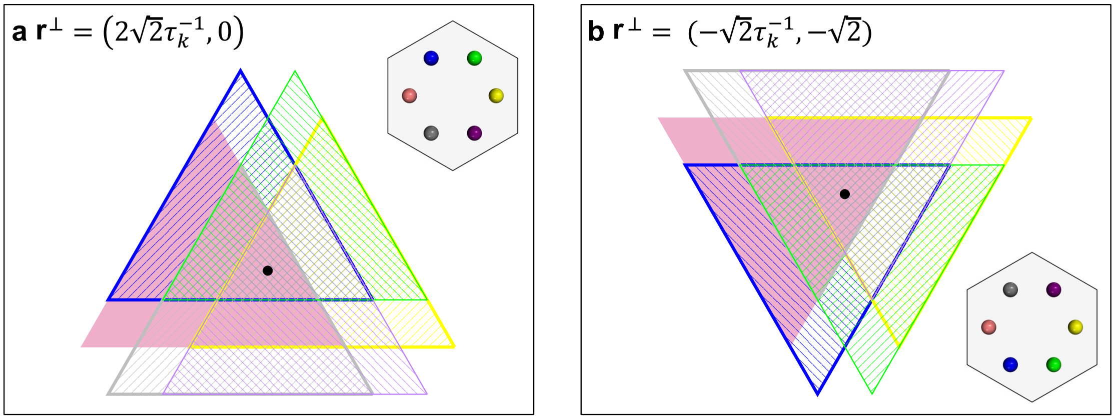

Supplementary Fig. 26. **Windows for the v group in the perpendicular space.** Windows for the $v^\triangle$ and $v^\triangledown$ groups in the perpendicular spaces with **a** $\mathbf{r}^\perp = (2\sqrt{2}\tau_k^{-1}, 0)$ and **b** $(-\sqrt{2}\tau_k^{-1}, -\sqrt{2})$ for the hexagonal golden-mean tiling with $k = 1$, respectively. Insets show the positions of atoms in the v group. Each layer has six distinct triangular windows, whose colors correspond to the colors of the atoms shown in the inset.

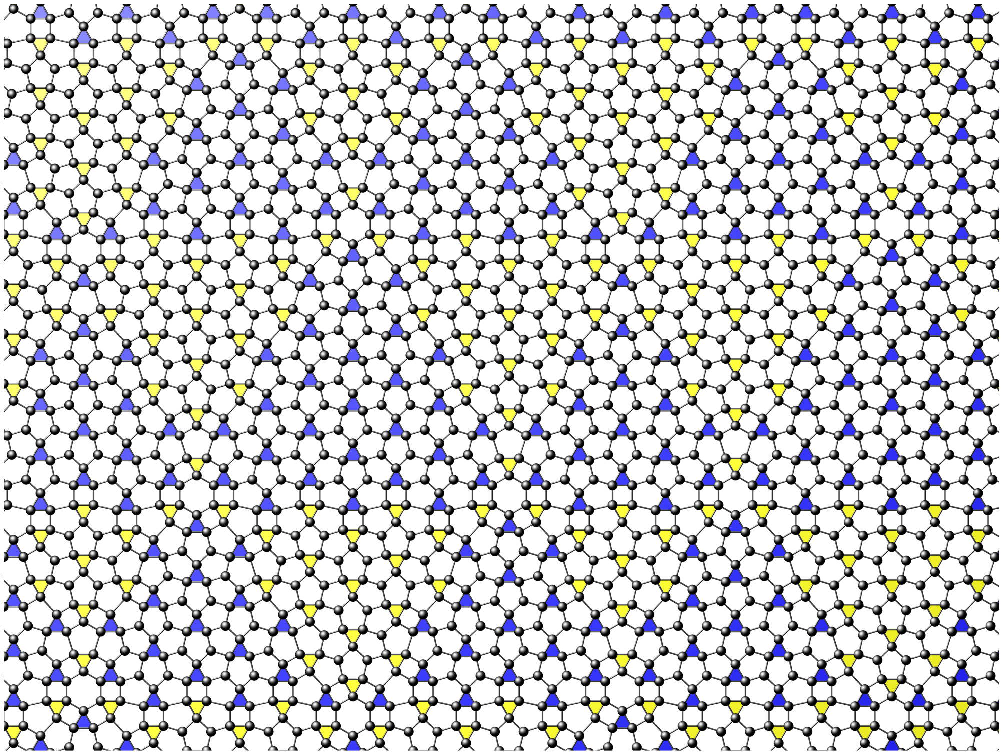

Supplementary Fig. 27. **Decorated hexagonal silver-mean tilings.**

We calculate the lattice structure factors of the decorated tilings, extending the method shown in Suppl. Note 7. The atoms in the decorated tiling are divided into three groups u_1, u_2, and v, as shown in Suppl. Fig. 24. The density of the atoms is then given as

$$\rho(\mathbf{r}) = \rho_{u_1}(\mathbf{r}) + \rho_{u_2}(\mathbf{r}) + \rho_v(\mathbf{r}), \tag{55}$$

$$\rho_{u_1}(\mathbf{r}) = \sum_{n=0}^{2}\left[\left(\sum_{j\in(0,0)^\perp} + \sum_{j\in(1,0)^\perp}\right)\delta\left(\mathbf{r}_j - c\mathbf{e}_n - \mathbf{r}\right)\right] + \sum_{n=0}^{2}\left[\left(\sum_{j\in(0,1)^\perp} + \sum_{j\in(1,1)^\perp}\right)\delta\left(\mathbf{r}_j + \mathbf{e}_n - \mathbf{r}\right)\right], \tag{56}$$

$$\rho_{u_2}(\mathbf{r}) = \sum_{n=0}^{2}\left[\sum_{j\in(2,0)^\perp}\delta\left(\mathbf{r}_j - c\mathbf{e}_n - \mathbf{r}\right)\right] + \sum_{n=0}^{2}\left[\sum_{j\in(-1,1)^\perp}\delta\left(\mathbf{r}_j + c\mathbf{e}_n - \mathbf{r}\right)\right], \tag{57}$$

$$\rho_v(\mathbf{r}) = \sum_{n=0}^{5}\left[\left(\sum_{j\in(2,0)^\perp} + \sum_{j\in(-1,1)^\perp}\right)\delta\left(\mathbf{r}_j + R^n\left\{\frac{\mathbf{e}_1 - \mathbf{e}_0}{3}\right\} - \mathbf{r}\right)\right], \tag{58}$$

where $\mathbf{r}_i$ represents the position of the vertex site in the tiling and the operator R rotates the vectors by the angle $\pi/3$, as

$$R = \begin{pmatrix} \cos(\pi/3) & -\sin(\pi/3) \\ \sin(\pi/3) & \cos(\pi/3) \end{pmatrix}. \tag{59}$$

The operation is represented as $R\mathbf{e}_i = -\mathbf{e}_j$ $(i = 0, 1, 2)$, where $j = i - 1 + 3\delta_{0i}$. We have used the position of atoms, which are explicitly shown in Suppl. Table 3. In the lattice structure factors, the peak positions are represented as $\mathbf{q} = \sum_{m=0}^{5}\tilde{n}_m\mathbf{q}_m$, where $\tilde{n}_m$ is an integer and $\mathbf{q}_m$ is the reciprocal vector derived in Suppl. Note 5. The Fourier transform of the density $\rho(\mathbf{q}) = \int \rho(\mathbf{r})\exp(-\mathrm{i}\mathbf{r}\cdot\mathbf{q})d\mathbf{r}$ can be represented in terms of the Fourier transform of the occupation domains $\mathcal{F}_{mn}(\tilde{\mathbf{q}})$ as,

$$\begin{aligned}\rho(\mathbf{q}) = {} & \exp(-\mathrm{i}\sqrt{2}c\tau_k^{-1}q_0^\perp)\sum_{n=0}^{2}\exp(-\mathrm{i}c\tilde{\mathbf{e}}_n\cdot\tilde{\mathbf{q}})\Big[\mathcal{F}_{00} + \exp(\mathrm{i}\sqrt{2}\tau_k^{-1}q_0^\perp)\mathcal{F}_{10} + \exp(\mathrm{i}2\sqrt{2}\tau_k^{-1}q_0^\perp)\mathcal{F}_{20}\Big] \\ & + \exp(\mathrm{i}\sqrt{2}c\tau_k^{-1}q_0^\perp)\sum_{n=0}^{2}\exp(\mathrm{i}c\tilde{\mathbf{e}}_n\cdot\tilde{\mathbf{q}})\exp(-\mathrm{i}\sqrt{2}q_1^\perp)\Big[\mathcal{F}_{01} + \exp(\mathrm{i}\sqrt{2}\tau_k^{-1}q_0^\perp)\mathcal{F}_{11} + \exp(-\mathrm{i}\sqrt{2}\tau_k^{-1}q_0^\perp)\mathcal{F}_{-11}\Big] \\ & + \sum_{n=0}^{5}\exp\left(\mathrm{i}R^n\left\{\frac{\tilde{\mathbf{e}}_1 - \tilde{\mathbf{e}}_0}{3}\right\}\cdot\tilde{\mathbf{q}}\right)\Big[\exp(\mathrm{i}2\sqrt{2}\tau_k^{-1}q_0^\perp)\mathcal{F}_{20} + \exp(-\mathrm{i}\sqrt{2}\tau_k^{-1}q_0^\perp)\exp(-\mathrm{i}\sqrt{2}q_1^\perp)\mathcal{F}_{-11}\Big].\end{aligned} \tag{60}$$

Then, we obtain the lattice structure factors $|\rho(\mathbf{q})|^2$.

Suppl. Fig. 28 shows the decorated tilings and the corresponding lattice structure factors for $k = 3$ and $k = 5$. We confirm that the peak structures of the decorated tilings are in a good agreement with the MC results shown in Suppl. Fig. 19.

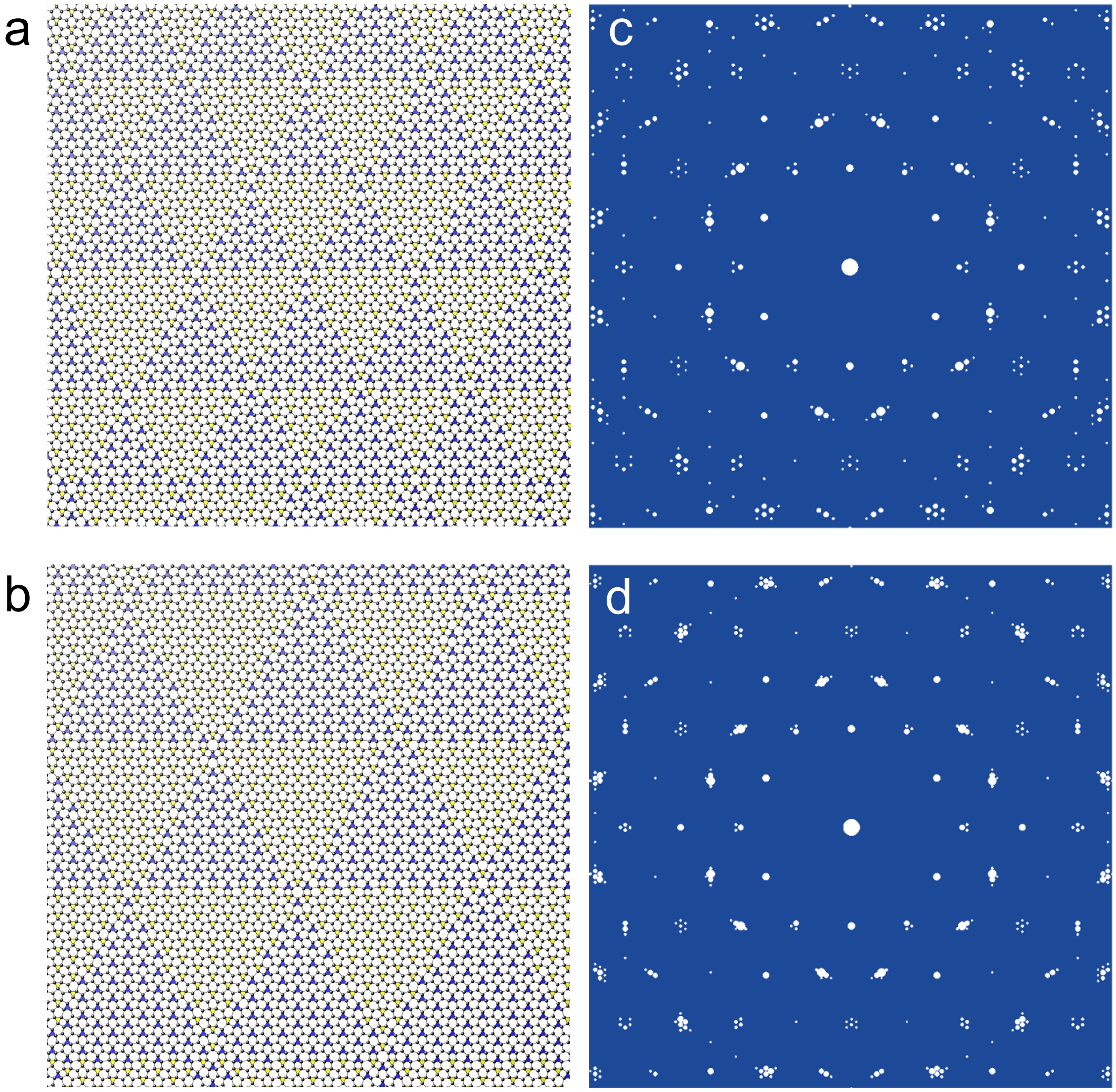

Supplementary Fig. 28. **The decorated tilings** (**a** $k = 3$ and **b** $k = 5$) **and the lattice structure factors** (**c** $k = 3$ and **d** $k = 5$). The area is proportional to the intensity. Source data are provided as a Source Data file.

Supplementary References

[1] Coates, S. *et al.* Hexagonal and trigonal quasiperiodic tilings (2023). arXiv:2201.11848.

[2] Engel, M. Entropic stabilization of tunable planar modulated superstructures. *Phys. Rev. Lett.* **106**, 095504 (2011).

[3] Engel, M. & Trebin, H.-R. Self-assembly of monatomic complex crystals and quasicrystals with a double-well interaction potential. *Phys. Rev. Lett.* **98**, 225505 (2007).
